# Biosynthetic insights provided by unusual sesterterpenes from the medicinal herb *Aletris farinosa* †
†Dedicated to Prof. Sir Alan Battersby on the occasion of his 90^th^ birthday.
‡
‡Electronic supplementary information (ESI) available: Full computational details, ^1^H and ^13^C NMR spectra for **2–6** and experimental procedures



**DOI:** 10.1039/c5sc02056e

**Published:** 2015-07-06

**Authors:** Victoria L. Challinor, Ryne C. Johnston, Paul V. Bernhardt, Reginald P. Lehmann, Elizabeth H. Krenske, James J. De Voss

**Affiliations:** a School of Chemistry and Molecular Biosciences , The University of Queensland , Brisbane 4072 , Queensland , Australia . Email: j.devoss@uq.edu.au; b Integria Healthcare Pty. Ltd. , Brisbane 4133 , Queensland , Australia

## Abstract

A series of novel sesterterpenes (**2–6**) have been isolated from the roots of *Aletris farinosa* and structurally characterized by MS, NMR, and X-ray crystallography in conjunction with computational modeling. Their structures provide new insights into the mechanisms of sesterterpene biosynthesis. Specifically, we propose with support from density functional theory computations that the configuration at a single stereocenter determines the fate of a key tetracyclic carbocationic intermediate, derived from an oxidogeranylfarnesol precursor. Whereas one epimer of the carbocation undergoes H^+^ elimination to give **6**, the other undergoes a spectacular cascade of seven 1,2-methyl and hydride migrations leading to the previously unreported carbon skeleton of **5**. Theoretical calculations suggest that the cascade is triggered by substrate preorganization in the enzyme active site.

## Abstract 2

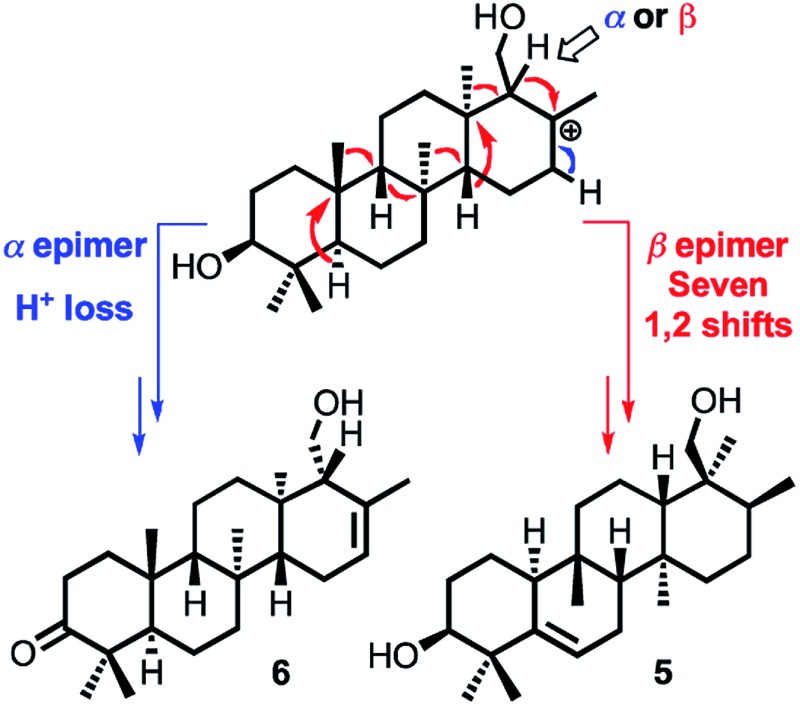
Configuration of a single stereocenter determines if a key carbocation in sesterterpene biosynthesis undergoes simple elimination or a cascade of seven 1,2-methyl and hydride migrations.

## Introduction

Sesterterpenes are a structurally diverse group of terpenoids, isolated most commonly from marine organisms, fungi and lichen.^1^ Little is known about the mechanisms of sesterterpene biosynthesis.^2^ Mono-, sesqui-, and Class I diterpene^3^ synthases initiate cyclisation by pyrophosphate ionization. Although analogous processes do explain the origin of some sesterterpenes,^2*a*^ it cannot always be assumed, as recent pioneering work on sesterterpene synthesis by squalene–hopene cyclases has demonstrated a different initiation mechanism involving protonation of the distal double bond of geranylfarnesol.^2*b*^ Herein, we report the unusual discovery of a suite of sesterterpenes from a higher plant, *Aletris farinosa*, that reveal important insights into this problem. The methylation patterns of this relatively simple family are highly informative, and the stereochemical relationships among the series provide evidence for conformation-determined control of the fate of the carbocation precursors by the enzyme.^4^ By use of quantum chemical calculations we show how the configuration at a single stereocenter determines whether the carbocation undergoes simple proton loss or an unprecedented cascade of seven 1,2-hydride and methyl migrations.

**Chart 1:**
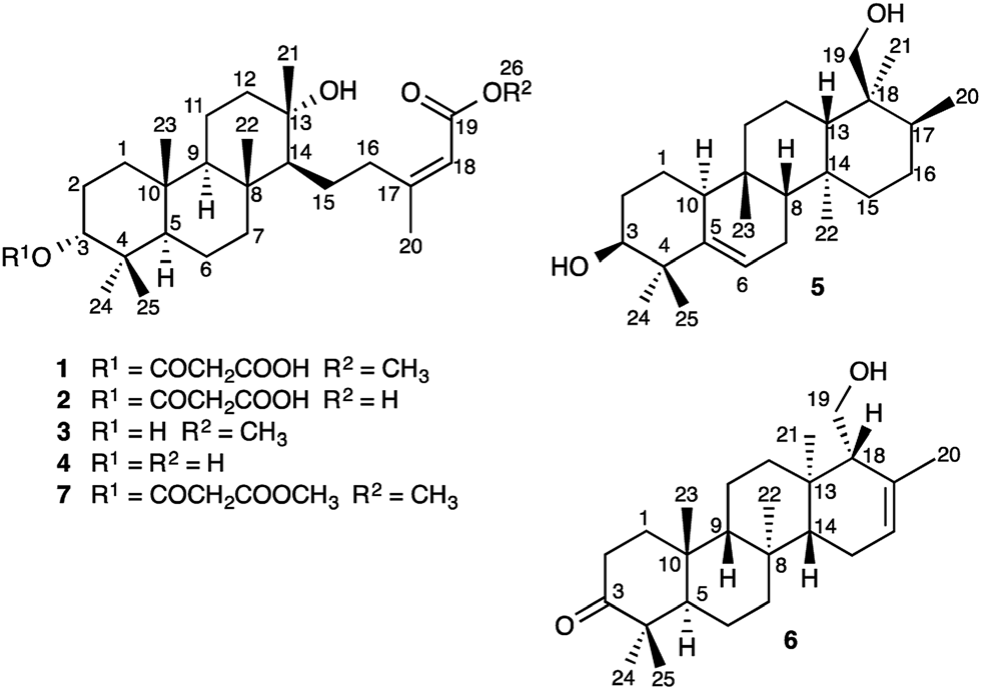
Structures of Sesterterpenes **1–7**.

*A. farinosa* L., or “true unicorn”, is a North American herb used in traditional medicine to treat inflammation, indigestion, colic, and women's reproductive health problems.^5^ The common name of this plant hints at its morphological similarity to “false unicorn”, *Chamaelirium luteum* (L.) A. Gray, which has an overlapping geographical distribution and has been used interchangeably with *A. farinosa* in herbal medicines.^6^ The phytochemistry of *C. luteum* is characterized by steroidal glycosides with structures closely related to cholesterol.^7^ Limited early literature suggested that these may also be present in *A. farinosa*.^8^ However, we recently discovered, unexpectedly, that the major constituent of *A. farinosa* is the unusual cheilanthane sesterterpene derivative **1** (Chart 1).^9^ Our efforts to elucidate the complete phytochemical profile of *A. farinosa* have now delivered three new analogs of **1** (**2–4**),^10^ and two novel tetracyclic sesterterpenes **5** and **6** with previously unreported carbon skeletons. Indeed it was clear that significant rearrangement must occur to deliver **5** from the usual terpene precursors. The structural relationship of these compounds coupled with theoretical calculations provide insight into the biosynthetic control of sesterterpene biogenesis.

## Results and Discussion

### Elucidation of the planar structures of **1–5**

Sesterterpenes **1–6** were extracted from the roots of *A. farinosa* and purified *via* solid phase extraction followed by HPLC. The structures of **2–4**, closely analogous to the recently reported cheilanthane **1**, were assigned by 1D and 2D NMR spectroscopy and, in the case of **2**, by chemical correlation with the known **7**.^9^ Compound **5**, an amorphous solid ([*α*]_D_ +32, *c* 0.23, CHCl_3_), gave a molecular formula of C_25_H_42_O_2_ (five degrees of unsaturation) by HRESIMS. The ^1^H NMR spectrum displayed six methyl group signals that, along with the molecular formula, were suggestive of a sesterterpene metabolite. The spectrum also displayed signals for olefin (*δ*_H_ 5.62 ppm, H-6), hydroxymethyl (*δ*_H_ 3.60, 3.70, and 5.75 ppm, H_2_-19 and 19-OH), and oxymethine groups (*δ*_H_ 3.74 and 5.40 ppm, H-3 and 3-OH). The ^13^C NMR spectrum displayed 25 signals, including peaks for two oxygenated carbons (*δ*_C_ 69.6 and 76.0 ppm, C-19 and C-3, respectively) and two olefinic carbons (*δ*_C_ 119.3 and 143.5 ppm, C-6 and C-5, respectively).

The long-range HMBC correlations of the methyl groups allowed assignment of key structural fragments within **5**. For example, the correlations of *δ*_H_ 1.14 (H_3_-24) and 1.42 ppm (H_3_-25) with C-3, C-4, and C-5 revealed at once the C-4 *gem*-dimethyl moiety, C-3 hydroxylation, and the Δ^5(6)^ double bond. The tetracarbocyclic backbone of **5** was completely assigned in an iterative manner *via* examination of COSY, TOCSY, HSQC, and HMBC spectra (Table 1, ESI‡), revealing a previously unreported sesterterpene skeleton with an unexpected and unique pattern of methylation.

### Determination of the stereochemistry of **5**

**Fig. 1:**
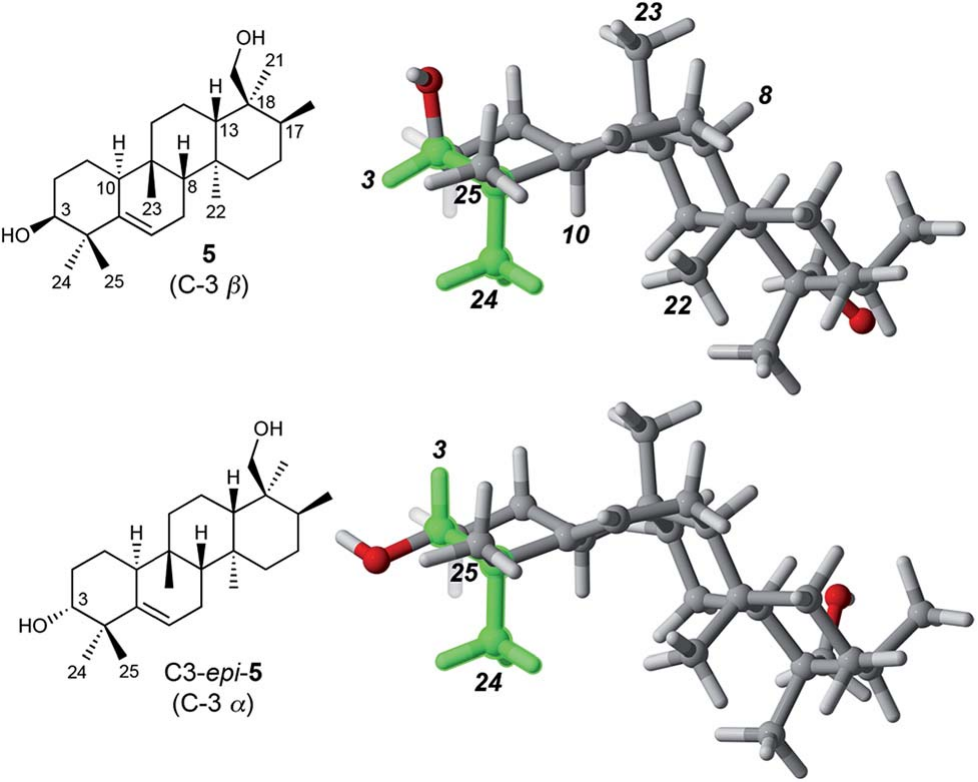
Preferred conformations of **5** and its C-3 epimer in pyridine, calculated at the PCM/B3LYP/6-31G(d,p) level of theory. The green shading highlights the *cis* relationship between H-3 and H_3_-24 in **5** and the *trans*-diaxial relationship in C3-*epi*-**5**. Only **5** is expected to show an NOE between H-3 and H_3_-24.

Determination of the relative stereochemistry of **5** hinged upon the NOESY correlations involving H-3 (Fig. 1). Cross-peaks between H-3 (*δ*_H_ 3.74) and both H_3_-24 and H_3_-25 suggested that H-3 occupies an equatorial position on a chair-like A ring, and thus that the C-3 hydroxyl group is axial (β as drawn). However, the unusual stereochemistry of the ring junctions (*vide infra*) led us to consider that the observed pair of cross-peaks between H-3 and H_3_-24/H_3_-25 might alternatively be consistent with C-3 α stereochemistry if the A ring in the C3 α epimer adopted a boat conformation. Density functional computations on the preferred conformations of the two epimers in pyridine with PCM/B3LYP/6-31G(d,p)^11^ indicated that the A ring favors a chair conformation in both epimers. The chair–boat energy difference (Δ*G*) is 1.2 kcal mol^–1^ for the β epimer and 3.8 kcal mol^–1^ for the α epimer. Thus, the C-3 α epimer of **5** should exist exclusively with the A-ring in a chair conformation, in which H-3 and H_3_-24 are *trans*-diaxial and would be unlikely to give rise to an NOE correlation. On this basis we assigned the configuration at C-3 in **5** as β.

Correlations between H_3_-24 (*δ*_H_ 1.14) and both H-10 (*δ*_H_ 2.51) and H_3_-22 (*δ*_H_ 1.04) showed that H-10 and H_3_-22 occupy the α face of the molecule. A strong correlation between H_3_-23 (*δ*_H_ 0.96) and H-8 (*δ*_H_ 1.13), and the absence of any correlations of these protons with H_3_-22 or H-10, indicated *cis* fusion of the B and C rings, with H_3_-23 and H-8 both occupying the β face. All of these correlations are consistent with the geometry of the predominant conformer calculated by DFT (Fig. 1).

Unambiguous stereochemical assignment at the remaining stereocenters C-13, C-17, and C-18 was prevented by signal overlap in the region of H-13 (*δ*_H_ 1.48) and H_3_-21 (*δ*_H_ 1.14). More sophisticated NMR experiments such as HSQC-NOESY were limited by sensitivity, and attempts to grow crystals suitable for X-ray analysis were unsuccessful. We therefore utilized computational methods to assign the relative stereochemistry at C-13, C-17, and C-18. The ^1^H and ^13^C NMR shielding constants for the eight diastereomers at these positions were computed at the PCM/mPW1PW91/6-311+G(2d,p)//PCM/B3LYP/6-31G(d,p) level of theory^11^ in pyridine. Then, the correlation coefficients between the computed and experimental chemical shifts for the different diastereomers were compared according to the method of Tantillo.^12^ The ^1^H correlation coefficients provided a clear diagnostic, indicating that the stereochemistry at C-13 was that corresponding to a *trans* C/D ring junction. All of the *cis*-fused diastereomers gave poor fit (*R* ^2^ ≤ 0.88) compared to the *trans*-fused isomers (*R* ^2^ ≥ 0.93). Among the four possible C-17, C-18 diastereomers containing the *trans* C/D junction, the best fit between computed and experimental ^1^H and ^13^C NMR shifts was afforded by the 13S*,17S*,18S* diastereomer, followed by 13S*,17S*,18R*. The ^13^C mean unsigned error (MUE) in the region of interest (C-13 & C-17–C-21) was also found to be diagnostic, with 13S*,17S*,18S* (MUE 1.46 ppm) less than half that of the 13S*,17S*,18R* diastereomer (MUE 3.07 ppm).

Separately, evaluation of the DP4 probabilities of the eight diastereomers according to Goodman's prediction formula^13^ provided clear independent confirmation of the assignment of configuration. DP4 calculations predicted the 13S*,17S*,18S* stereochemistry with a probability of 99.8%.^12^

### Elucidation of the planar structure of **6**

Compound **6** was obtained as an amorphous solid ([*α*]_D_ + 64, *c* 0.12, CHCl_3_) and HRESIMS provided a molecular formula of C_25_H_40_O_2_ (six degrees of unsaturation). The ^1^H NMR spectrum displayed signals for six methyl groups, in addition to peaks for an olefinic proton (*δ*_H_ 5.57 ppm, H-16) and a hydroxymethyl group (*δ*_H_ 3.93, 4.11, and 5.74 ppm, H_2_-19, 19-OH). The ^13^C NMR spectrum displayed signals for one carbonyl (*δ*_C_ 218.8 ppm, C-3) and two olefinic carbons (*δ*_C_ 122.8 and 134.9 ppm, C-16 and C-17, respectively). In combination with COSY, TOCSY, and HSQC spectra, interpretation of the HMBC correlations of the methyl groups allowed complete assignment of the tetracarbocyclic skeleton (Table 1, ESI‡), providing the planar structure of **6** (Chart 1).

### Determination of the stereochemistry of **6**.

**Scheme 1:**
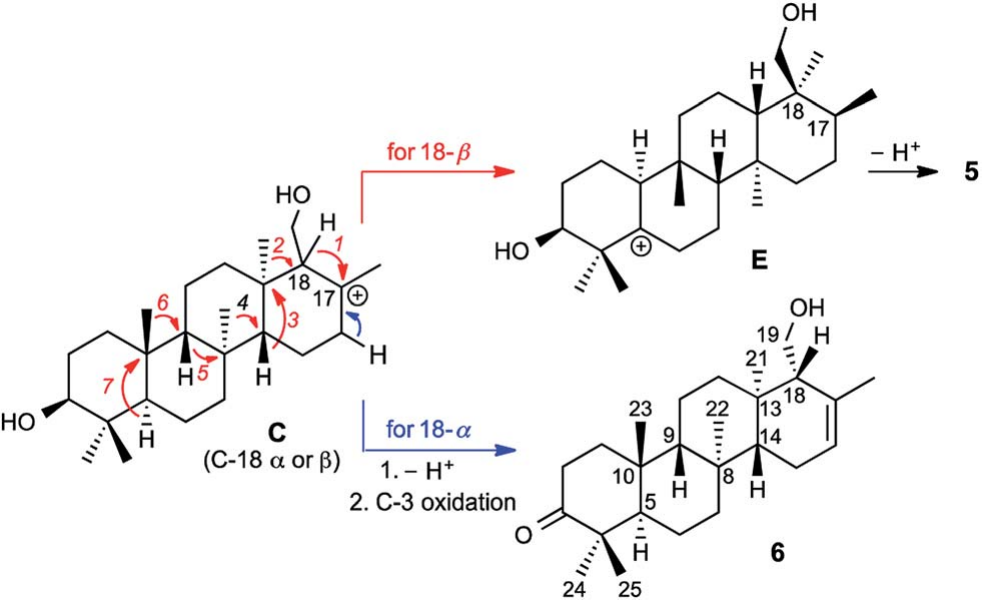
Proposed biosynthetic and stereochemical relationships between **5** and **6**.

Prior to determination of the relative stereochemistry of **6**, we noted that the methylation pattern of **6** suggested a biosynthetic relationship to **5**, and thus that there may be a stereochemical correlation between the two. We proposed that the unusual methylation pattern of **5** originates from a series of 1,2-hydride and methyl migrations in a tetracyclic carbocation that might also serve as precursor to **6** (**C**, Scheme 1). The proposed overall transformation to **5** is shown in Scheme 1 (red arrows). Starting from carbocation **C**, a sequence of seven suprafacial 1,2-hydride and methyl migrations would furnish carbocation **E**, the immediate precursor to **5**. The same carbocation **C** could also potentially provide the carbon skeleton of **6** *via* simple loss of H^+^ (Scheme 1, blue arrows).

The 2D NOESY spectrum of **6** supported the proposed stereochemical correlation, revealing direct relationships between the relative stereochemistry of **5** and **6** at all centers except C-18. Cross-peaks between H_3_-23 (*δ*_H_ 0.73) and H_3_-25 (*δ*_H_ 1.09), and between H_3_-24 (*δ*_H_ 1.06) and H-5 (*δ*_H_ 2.23), confirmed the *trans* **A**/**B** ring junction. A correlation between H_3_-23 and H-9 (*δ*_H_ 0.94) confirmed the *cis* relationship of these two groups. Correlations between H_3_-22 (*δ*_H_ 1.08) and H-5, and between H_3_-21 (*δ*_H_ 0.98) and H_3_-22, showed that the C-21 and C-22 methyl groups reside on the same (α) face of the molecule, while a correlation between H-9 and H-14 (*δ*_H_ 1.14) indicated that these protons reside on the β face, thereby confirming the **B**/**C** and **C**/**D** ring junctions to be *trans*. Thus, the stereochemistry of the ring junctions in **6** correlate with those seen in **5** as would be expected for biogenically related compounds.

However, correlations of H_2_-19 (*δ*_H_ 3.93 and 4.11) with H_3_-21 indicated that the hydroxymethyl at C-18 possesses α stereochemistry in **6**. In **5**, on the other hand, C-18 has β stereochemistry. This implies that they are not derived from a single carbocationic precursor but that the biosynthetic pathways leading to **5** and **6** diverge prior to the installation of the C-18 stereocenter: that is, prior to the closure of the **D** ring.

**Fig. 2:**
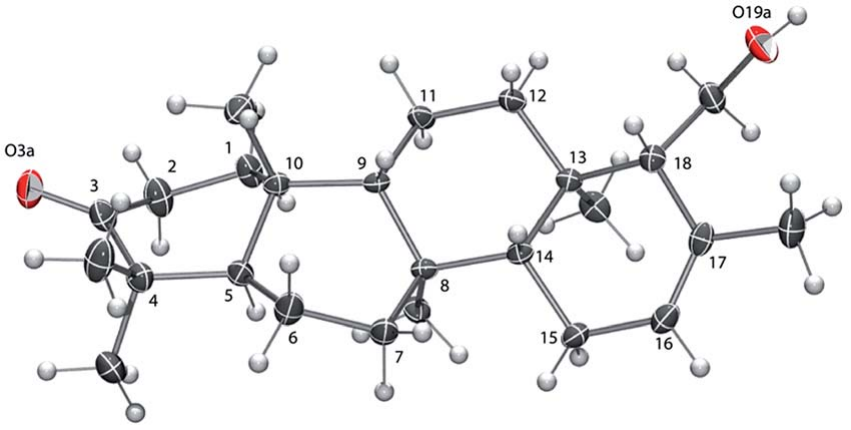
ORTEP view of **6** with ellipsoids drawn at 30% probability.^14^ The ring C-atom numbering is shown.

The absolute configuration of **6** was established as that shown in Chart 1 *via* X-ray crystallographic analysis. Suitable crystals were grown *via* slow evaporation of a methanol/water solution (Fig. 2). The X-ray structure^14^ agreed with the NMR-based assignment of relative stereochemistry and, in particular, confirmed the α configuration at C-18. Sesterterpene **6** therefore belongs to the same enantiomeric series as **1–4**, with identical absolute configuration at key centers (*e.g.* C-5 and C-10). The absolute configuration of **5** could not be conclusively proven. However, theoretical prediction of [*α*]_D_ by means of DFT calculations [B3LYP/aug-cc-pVDZ//B3LYP/6-31G(d)] (see the ESI‡) supported the assignment as belonging to the same enantiomeric series as **1–4** and **6**.

### Biosynthetic Implications.

The *A. farinosa* metabolites **1–6** represent three distinct sesterterpene classes: tricyclic compounds **2–4** related to the major cheilanthane **1**, and the divergent tetracyclic structures **5** and **6**. The differing stereochemistry at the **B**/**C** ring junctions eliminates the possibility that **6** is formed by one additional cyclization of the precursor to **1–4**. Differentiation of **5** and **6** from the tricyclic sesterterpenes **1–4** arises at an earlier stage of the biosynthesis. In general, it is not known if the cyclases responsible for the biosynthesis of sesterterpenes initiate cyclisation by pyrophosphate ionization (as in mono-, sesqui-, and Class I diterpene synthases)^2,15^ or by acid-catalyzed activation of an alkene or epoxide (as in triterpene synthases).^16^ Triterpene squalene–hopene cyclases have been shown to protonate a double bond of geranylfarnesol and thus catalyze sesterterpene formation.^2^ The presence of C-3 oxygenation in all of the isolated metabolites (**1–6**) makes it reasonable to postulate that further similarity between triterpene and sesterterpene biosynthesis exists and that ring opening of an oxidogeranylfarnesyl precursor initiates a cascade of cyclization reactions analogous to those catalyzed by 2,3-oxidosqualene cyclases.^16^ Alternatively, C-3 oxygenation could be introduced in a separate step after proton initiated cyclisation of a geranylfarnesyl precursor to give a tetracyclic intermediate.

**Fig. 3:**
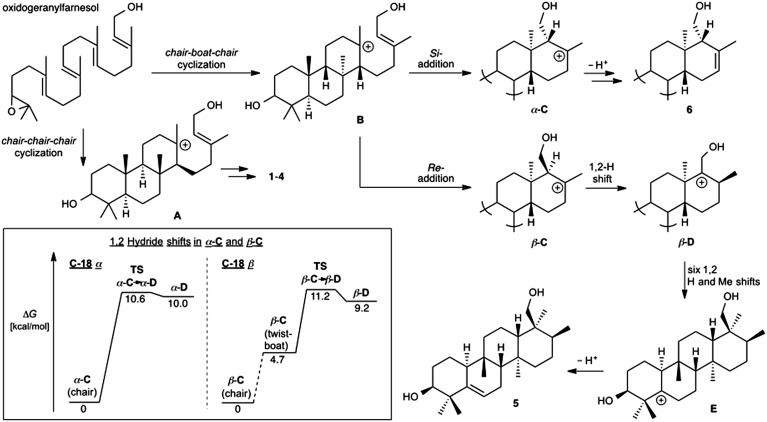
Proposed biosynthetic routes to sesterterpenes **1–4**, **5**, and **6**. The inset shows the computed free energy profiles for 1,2-hydride migrations in the C-18 epimeric carbocations α-**C** and β-**C**.

We propose that sesterterpenes **1–6** are formed by the biosynthetic pathways shown in Fig. 3. Folding of a geranylfarnesol precursor into a *chair*–*chair*–*chair* conformation, followed by tricyclization, would lead to cation **A** that is quenched by water to yield the cheilanthanes **1–4**. On the other hand, a *chair*–*boat*–*chair* mode of tricyclization, followed by **D** ring closure onto the *Si*- or *Re*-face of the olefin in intermediate **B**, would lead to carbocations α-**C** and β-**C**, respectively.^17^ Simple quenching of α-**C** by elimination of H^+^ leads to **6**, whereas β-**C** undergoes the cascade of seven hydride and methyl migrations (Scheme 1) terminating in H^+^ elimination and the formation of the Δ^5(6)^ double bond of **5**.

In this biosynthetic scheme, the routes to sesterterpenes **5** and **6** diverge when cation **B** cyclizes to **C**. The C-18 stereochemistry plays a crucial role, such that only β-**C** undergoes the C-18 → C-17 hydride shift that initiates the cascade of 1,2 migrations leading to **5**. Theoretical calculations reveal a possible scenario by which this divergence of biosynthetic fates may arise.^18^ Free energy profiles for C-18 → C-17 hydride migrations in α- and β-**C**, computed at the B3LYP/6-31+G(d,p) level of theory, are shown in the inset to Fig. 3. Interestingly, the barrier to hydride migration is about the same in both epimers (∼11 kcal mol^–1^). However, while α-**C** reacts as a chair conformer, hydride migration in β-**C** can occur only from a twist-boat conformation. This twist-boat conformer of β-**C** lies 4.7 kcal mol^–1^ above the chair.

This theoretical result suggests a possible mechanism by which the cascade of hydride and methyl shifts leading to **5** could be triggered. The reactive twist-boat conformer of β-**C** is generated when the cyclization of **B** takes place through a boat-like transition state to deliver the observed stereochemistry at C-18, and might then be prevented from converting into the chair by virtue of the three-dimensional features of the enzyme active site. Preorganization of β-**C** into the twist-boat conformation would reduce the barrier for the 1,2-hydride shift by about 5 kcal mol^–1^, corresponding to a theoretical rate enhancement of 10^3^. This mode of substrate activation is only possible for β-**C**. The epimeric cation α-**C**, in the absence of such activation, does not undergo 1,2-hydride shift and instead undergoes simple elimination of H^+^.^19^

To evaluate this biosynthetic hypothesis, we attempted to effect an acid-catalyzed rearrangement of **6**. Treatment of **6** with a proton source would generate cation α-**C**. Previously, Brownlie and coworkers^20^ reported the conversion of the triterpene friedelene to oleanene—a rearrangement involving a cascade of six hydride and methyl migrations—by exposure to acetic acid/hydrochloric acid under reflux. Reaction of **6** under these conditions did not, however, lead to rearranged products corresponding to 17-*epi*-**5**; GCMS analysis indicated that simple elimination of water had occurred instead.

## Conclusions

The isolation of the novel suite of co-occurring compounds **1–6** from *A. farinosa* has yielded important new insights into the mechanisms of sesterterpene biosynthesis. Oxygenation at C-3 suggests that **1–6** are biosynthesized from a common epoxide precursor – a feature more analogous to triterpene biogenesis than mono-, sesqui- or diterpene biogenesis. Experimental and theoretical evidence suggests that the biosynthesis of the previously unknown carbon framework of **5** involves an unprecedented sequence of seven 1,2-hydride and methyl shifts in a tetracyclic carbocation. Initiation of the cascade appears to be controlled by the stereochemistry at a single center, C-18, which provides a basis for conformationally-induced substrate activation by the biosynthetic enzyme. Hydrogen bonding between the enzyme and the C-18 hydroxymethyl represents a likely mode of stabilization of the requisite preorganized conformation. If this is the case, then replicating nature's sequence of migrations **C** → **E** in a laboratory synthesis of **5** is likely to be very difficult.
