# Supplementary material for: Biosynthetic insights provided by unusual sesterterpenes from the medicinal herb Aletris farinosa
Source: Chem Sci. 2015 Jul 6;6(10):5740–5. doi: 10.1039/c5sc02056e (PMC5633834; doi:10.1039/c5sc02056e)
Supplement: Supplementary file 1 [file SC-006-C5SC02056E-s001.pdf]

Electronic Supplementary Material (ESI) for Chemical Science.
This journal is © The Royal Society of Chemistry 2015

Biosynthetic Insights Provided by Unusual Sesterterpenes from the Medicinal Herb *Aletris farinosa*

Victoria L. Challinor, Ryne C. Johnston, Paul V. Bernhardt, Reginald P. Lehmann, Elizabeth H. Krenske, and James J. De Voss

Supplementary Information

General Experimental Procedures 3

Plant Material 3

Extraction and Isolation 3

Esterification of **2** to produce **7** 4

Esterification of **4** to produce **3** 5

Treatment of **6** with acid 5

X-ray Crystallographic Data for **6** 6

Figure S1. ORTEP view of **6** 6

Table S1. ^{1}H and ^{13}C NMR spectroscopic data for **2**, **3**, **5**, and **6** 7

^{1}H and ^{13}C NMR spectra of **2** 8

^{1}H and ^{13}C NMR spectra of **3** 9

^{1}H and ^{13}C NMR spectra of **4** 10

^{1}H and ^{13}C NMR spectra of **5** 11

^{1}H and ^{13}C NMR spectra of **6** 12

Computational Methodology 13

Preferred Conformation of the C3-α Epimer of **5** 13

Optimized geometries of conformers of C3-epi-**5** at the $PCM_{pyridine}$/B3LYP/6-31G(d,p) level of theory 14

General Notes on NMR Shift Calculations 19

Table S2. Scaling factors for the present level of theory 19

Table S3. Comparison of Computed NMR shifts for Each Diastereomer of **5** 20

Table S4. Comparison of Weighted Average Computed NMR shifts for (13*S**,17*S**,18*S**)-**5** 22

Table S5. Computed Chemical Shifts for (13*S**,17*S**,18*S**)-**5** 23

Molecular geometries of diastereomers of compound **5** at the $PCM_{pyridine}$/B3LYP/6-31G(d,p) level of theory 25

Theoretical Prediction of Optical Rotation of **5** 42

Figure S2. Comparison of experimental and calculated $[\alpha]_D$ values for the enantiomers of **5** 42

Optimized geometry of **5** at the B3LYP/6-31G(d) level of theory 43

Carbocation Rearrangements Leading to **5** and **6** 45

Optimized geometries at the B3LYP/6-31+G(d,p) level of theory 45

References 55

General Experimental Procedures

Optical rotations were measured on a JASCO P-2000 polarimeter. ^{1}H and ^{13}C NMR spectra were recorded on a Bruker AV400 or AV500 as indicated, with the residual protonated pyridine-d_5 (δ_H 8.71 ppm; δ_C 149.9 ppm) or chloroform (δ_H 7.24 ppm; δ_C 77.0 ppm) signal as internal standard. *J* values are expressed in Hz. Low-resolution mass spectra were recorded on a Bruker ESQUIRE HCT instrument (positive and negative-ion ESI). High-resolution mass spectra were recorded on a Bruker MicrOTOF-Q instrument (positive and negative-ion ESI) with internal calibration using Agilent Tune-Mix. RPHPLC was performed on a Shimadzu LC-10AT liquid chromatograph equipped with an ELSD-LT detector (52°C, N_2 pressure: 200 KPa), column oven (30°C), and a semi-preparative Phenomenex HPLC column (Luna C-18, 5 μm, 250 × 10 mm). GSMS spectra were recorded on a Shimadzu GCMS QP2010-plus spectrometer; ZB-5MS column – 30 m. Standard GCMS program: column flow 1.7 mL/min; total flow 66.3 mL/min; injector 250°C; detector 250°C; oven 200°C held for 1 min, increased to 300°C (20°C/min) and held for 44 min; total program time 50 min.

Plant Material

A voucher specimen of *A. farinosa* was collected in the Midlands Wildlife Area, Delaware, USA (September 2011), and has been deposited at the Medicinal Plant Herbarium, Southern Cross University, Lismore, Australia (accession number PHARM-110043), where it was identified by Dr. H. Wohlmuth.

Extraction and Isolation

Powdered roots of *A. farinosa* (23 g) were extracted (80% MeOH(aq), 250 mL) with sonication (60 min). Following filtration and removal of the solvent in vacuo, the crude extract was partially purified via solid-phase extraction (Phenomenex Strata C-18E cartridge) eluting with H_2O followed by MeOH. The combined MeOH fractions were concentrated in vacuo, dissolved (90% MeOH(aq),

10 mL), filtered, and purified by semipreparative RPHPLC (gradient of 20% to 100% aqueous CH_3CN over 60 min, 2 mL/min). Five different fractions were collected, numbered I-V in order of elution. Fractions I-III were pure and corresponded to compounds **2** (t_R = 47.7 min, 19.8 mg) and **4** (t_R = 50.4 min, 10.1 mg) and **1**[1] (t_R = 57.0 min, 79 mg). Fraction IV (t_R = 59.4 min) was subjected to further purification via semipreparative RPHPLC (isocratic conditions of 75% aqueous CH_3CN over 40 min) to yield **5** (t_R = 35.5 min, 2.8 mg). Fraction V (t_R = 61.2 min) was subjected to further purification via semipreparative RPHPLC (isocratic conditions of 70% aqueous CH_3CN over 20 min) to yield **3** (t_R = 53.9 min, 3.1 mg) and **6** (t_R = 56.2 min, 1.9 mg). Compounds **1-4** have been previously reported in the patent literature,[2] albeit with differing, apparently erroneous, stereochemistry of the $\Delta^{17(18)}$ double bond. Compounds **1**, **2**, and **3** display ^{1}H and ^{13}C NMR chemical shifts that are in good agreement with the values reported by Takikawa and coworkers;[2] compound **4** can be converted to **3** via esterification (*vide infra*), confirming their structural relationship.

Compound 2: amorphous solid; $[\alpha]^{25}_D$ – 26 (*c* 0.15, $CHCl_3$); ^{1}H (pyridine-d_5, 400 MHz) and ^{13}C NMR (pyridine-d_5, 100 MHz), see Table 1; positive-ion ESIMS *m*/*z* 515; negative-ion ESIMS *m*/*z* 491; HRESIMS *m*/*z* 491.3005 $[M - H]^-$ (calcd for $C_{28}H_{43}O_7$, 491.3014). The ^{1}H NMR (chloroform-*d*, 400 MHz) chemical shifts of **2** were in close agreement with those reported for the corresponding planar structure ($\Delta\delta_H \leq 0.02$ ppm for selected key shifts).[2] For ^{13}C NMR (chloroform-*d*, 100 MHz), the observed peaks (resonances for C1′-C-3′ and C-19 were not detected in our ^{13}C NMR experiments) were in good agreement with those reported ($\Delta\delta_C \leq 0.3$ ppm), with the exception of C-13 (δ_C 73.1), which appeared 1.3 ppm upfield of the literature value (δ_C 74.4).[2]

Esterification of 2 to produce 7: Compound **2** (2 mg) was dissolved in MeOH (1 mL) before addition of ethereal CH_2N_2 (500 µL). The solution was stirred at 25°C for 30 min, before concentration to dryness under a stream of nitrogen, yielding a compound with identical ^{1}H and ^{13}C NMR spectra to those of diester **7** derived from naturally occurring **1**.[1]

Compound 3: amorphous solid; $[\alpha]_D - 32$ (c 0.26, $CHCl_3$); ^1H (pyridine-d_5, 500 MHz) and ^{13}C NMR (pyridine-d_5, 125 MHz), see Table 1; positive-ion ESIMS *m*/*z* 443; HRESIMS *m*/*z* 443.3136 $[M + Na]^+$ (calcd for $C_{26}H_{44}NaO_4$, 443.3132). The ^1H (chloroform-*d*, 500 MHz) and ^{13}C NMR (chloroform-*d*, 125 MHz) chemical shifts of **3** were in close agreement ($\Delta\delta_H \leq 0.06$ ppm for selected key shifts, $\Delta\delta_C \leq 0.2$ ppm) with those reported for the corresponding planar structure.[2]

Compound 4: amorphous solid; $[\alpha]_D - 26$ (c 0.38, $CHCl_3$); ^1H (pyridine-d_5, … MHz) and ^{13}C NMR (pyridine-d_5, 100 MHz); positive-ion ESIMS *m*/*z* 429; negative-ion ESIMS *m*/*z* 405; HRESIMS *m*/*z* 405.3001 $[M - H]^-$ (calcd for $C_{25}H_{41}O_4$, 405.3010). ^1H NMR (pyridine-d_5, 500 MHz) δ_H 6.06 (s, 1H), 3.60 (t, J = 2.6 Hz, 1H), 3.21 (m, 1H), 2.90 (m, 1H), 1.97-2.06 (m, 3H), 1.92 (d, J = 1.1 Hz, 3H, H_3-20), 1.34 (s, 3H, H_3-21), 1.18-1.88 (m, 11H), 1.17 (s, 3H, H_3-24), 1.05-1.12 (m, 1H), 0.89 (s, 3H, H_3-25), 0.85 (s, 7H, H_3-22 and H_3-23); ^{13}C NMR (pyridine-d_5, 125 MHz) δ_C 170.3, 158.8, 118.8, 75.3, 73.4, 62.5, 60.6, 49.3, 44.6, 41.6, 39.7, 38.1, 37.7, 37.6, 33.7, 29.3, 26.6, 25.4, 24.8, 24.6, 22.4, 19.7, 18.2, 17.0, 16.5.

Esterification of 4 to produce 3: Compound **4** (2 mg) was dissolved in MeOH (1 mL) before addition of ethereal CH_2N_2 (500 μL). The solution was stirred at 25°C for 30 min, before concentration to dryness under a stream of nitrogen, yielding a compound with identical ^1H and ^{13}C NMR spectra to those of **3**.

Compound 5: amorphous solid; $[\alpha]_D + 32$ (c 0.23, $CHCl_3$); ^1H (pyridine-d_5, 500 MHz) and ^{13}C NMR (pyridine-d_5, 125 MHz), see Table 1; positive-ion ESIMS *m*/*z* 397; negative-ion ESIMS *m*/*z* 373; HRESIMS *m*/*z* 397.3073 $[M + Na]^+$ (calcd for $C_{25}H_{42}NaO_2$, 397.3077).

Compound 6: amorphous solid; $[\alpha]_D + 64$ (c 0.12, $CHCl_3$); ^1H (pyridine-d_5, 500 MHz) and ^{13}C NMR (pyridine-d_5, 125 MHz), see Table 1; positive-ion ESIMS *m*/*z* 395; negative-ion ESIMS *m*/*z* 387; HRESIMS *m*/*z* 395.2921 $[M + Na]^+$ (calcd for $C_{25}H_{40}NaO_2$, 395.2921).

Treatment of 6 with acid: Using a protocol adapted from Brownlie et al.,[3] a solution of **6** (1mg) in acetic acid (2.4 mL) and conc. HCl (600 μL) was heated under reflux for 18 h before cooling to rt. To a sample of the reaction mixture (500 μL)

was added DCM (5 mL) and saturated sodium bicarbonate (5 mL). The organic layer was dried over sodium sulfate and concentrated under a stream of nitrogen to a volume of approximately 100 μL. GCMS analysis indicated that the major product had undergone elimination of water (**6**: t_R = 9.0 min, *m/z* 372; Major product: t_R = 6.1 min, *m/z* 354).

X-ray Crystallographic Data for 6

$C_{25}H_{40}O_2$, M_r 372.59, specimen 0.20 × 0.20 × 0.15 mm, monoclinic, $P2_1$ (No. 4), *a* 14.0700(5), *b* 10.4060(3), *c* 14.8885(4) Å, β 103.249(3)°, *V* 2121.8(1) Å^3, *Z* 4, ρ_{calc} 1.166 g cm^{-3}, μ 0.543 mm^{-1}, Cu-Kα λ 1.5418 Å, *T* 190 K, $2\theta_{max}$ 125°, N_{total} 31189, N_{unique} 6711, N_{obs} ($I > 2\sigma$) 6365, R_{int} 0.0365, *R* (obs. data) 0.0446, wR_2 (all data) 0.1279, residual electron density +0.83 and -0.33 e Å^{-3}. Data were acquired on an Oxford Diffraction Gemini CCD diffractometer with the crystal cooled by an Oxford Cryosystems Desktop Cooler. The structure was solved by direct methods with SHELXS and refined by full matrix least squares analysis with SHELXL97[4] within the WinGX interface.[5] The structure diagram was drawn with ORTEP3[6] and rendered with PovRay (version 3.6). The absolute configuration was established by the analysis of 3128 Bijvoet pairs according to the method of Hooft *et al.*[7] The probability of the correct enantiomer *P*2 was 1.00, with a Hooft parameter 0.2(1) (*v* = 15, Student's *t* statistics). Data in CIF format have been deposited with the Cambridge Crystallographic Data Centre (CCDC No. 1000114).

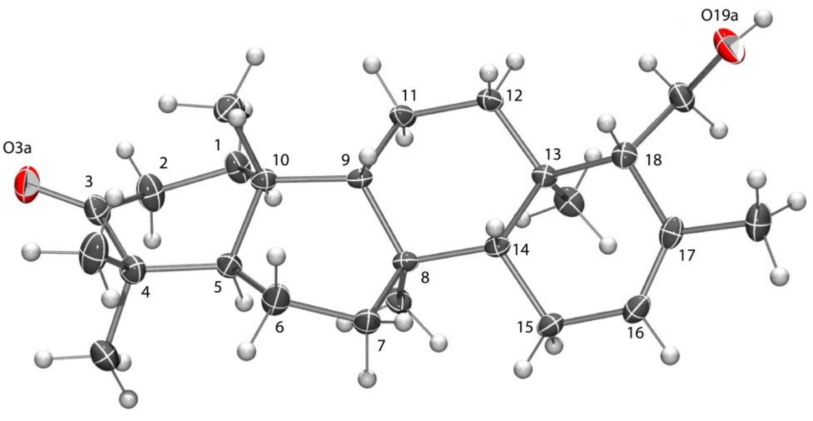

Figure S1. ORTEP view of **6** (30% probability ellipsoids). The ring C-atom numbering is shown.

Table S1. ^{1}H and ^{13}C NMR spectroscopic data for compounds **2**, **3**, **5**, and **6**.

	**2**[a]		**3**[a]		**5**[b]		**6**[b]	
Position	^{1}H [δ, mult, J (Hz)]	^{13}C	^{1}H [δ, mult, J (Hz)]	^{13}C	^{1}H [δ, mult, J (Hz)]	^{13}C	^{1}H [δ, mult, J (Hz)]	^{13}C
1a	1.30-1.44 m[c]	34.1	1.70 m[c]	33.7	2.00 m[c]	21.0	1.95 m[c]	31.7
1b	1.30-1.44 m[c]		1.45 m[c]		1.60 m		1.37 m[c]	
2a	1.79-1.92 m[c]	23.2	2.02 m[c]	26.6	1.91-2.04 m[c]	30.1	2.72 ddd (15.7, 11.9, 6.0)	33.9
2b	1.79-1.92 m[c]		1.78 m		1.91-2.04 m[c]		2.32 ddd (15.7, 9.7, 3.0)	
3	4.93 br s	79.2	3.60 m[c]	75.3	3.74 m	76.0		218.8
4		37.1		38.1		41.6		47.0
5	1.48 m[c]	50.6	1.71 m[c]	49.2		143.5	2.23 dd (13.1, 2.0)	45.1
6a	1.31-1.44 m[c]	18.0	1.53 m	18.3	5.62 m	119.3	1.35 m[c]	19.1
6b	1.31-1.44 m[c]		1.44 m[c]				1.23 m[c]	
7a	2.00 m[c]	41.3	2.06 m[c]	41.7	2.29 m	22.9	1.33-1.45 m[c]	39.7
7b	1.17 m		1.21 m		2.08 dd (19.0, 5.8)		1.33-1.45 m[c]	
8		39.6		39.7	1.13 m[c]	55.9		36.9
9	1.03 m[c]	60.5	1.09 m	60.7		35.8	0.94 dd (11.0, 3.8)	52.1
10		37.5		37.7	2.51 m	37.3		36.1
11a	1.52 m[c]	19.6	1.64 m[c]	19.7	2.01 m[c]	38.5	1.35 m[c]	19.1
11b	1.24 m[c]		1.29 m[c]		1.13 m[c]		1.35 m[c]	
12a	1.97 m[c]	44.4	2.01 m[c]	44.8	1.38-1.49 m[c]	19.1	2.18 dt (12.8, 3.2)	41.1
12b	1.66 m[c]		1.70 m[c]		1.38-1.49 m[c]		1.17 m[c]	
13		73.3		73.2	1.48 m[c]	47.0		36.9
14	1.38 m	62.5	1.38 t (3.9)	62.8		39.0	1.14 m[c]	56.5
15a	1.88 m[c]	24.8	1.85 m	24.5	1.49 m[c]	34.9	1.99 m[c]	23.2
15b	1.64 m[c]		1.60 m[c]		1.15 m[c]		1.88 m[c]	
16a	3.11 m	37.6	3.04 td (11.9, 5.8)	37.7	1.99 m[c]	26.2	5.57 m	122.8
16b	2.96 m		2.94 td (11.7, 4.7)		1.34 m[c]			
17		160.4 br		162.5	1.93 m[c]	35.7		134.9
18	6.04 br s	117.6 br	5.77 d (1.1)	115.5		39.8	2.09 m[c]	58.3
19a		*n.d.*		166.9	3.70 dd (10.2, 4.4)	69.6	4.11 dt (10.8, 3.1)	60.1
19b					3.60 m		3.93 dt (10.7, 5.3)	
20	1.93	25.5	1.90 d (1.2)	25.3	1.27 d (7.1)	15.7	2.08 br s	22.5
21	1.32 s	24.7	1.34 s	24.5	1.14 s	20.2	0.98 s	15.5
22	0.81 s	17.0	0.87 s	17.0	1.04 s	16.5	1.08 s	21.7
23	0.77 s	16.2	0.86 s	16.5	0.96 s	28.7	0.73 s	22.7
24	0.99 s	28.2	1.17 s	29.3	1.14 s	28.2	1.06 s	29.1
25	0.83	21.8	0.89 s	22.4	1.42 s	26.4	1.09 s	19.8
26			3.61 s	50.8				
1′		*n.d.*						
2′	*n.d.*	*n.d.*						
3′		*n.d.*						

[a]Acquired in pyridine-d_5 (500 MHz for ^{1}H and 100 MHz for ^{13}C).
[b]Acquired in pyridine-d_5 (500 MHz for ^{1}H and 125 MHz for ^{13}C).
[c]Indicates overlapping signals within column.
n.d. Signals for the carboxylate (C-19) and malonate (C-1′-C-3′) moieties were not detected, however esterification of **2** yielded a compound with identical spectroscopic properties to those of previously reported diester **7**.[1]

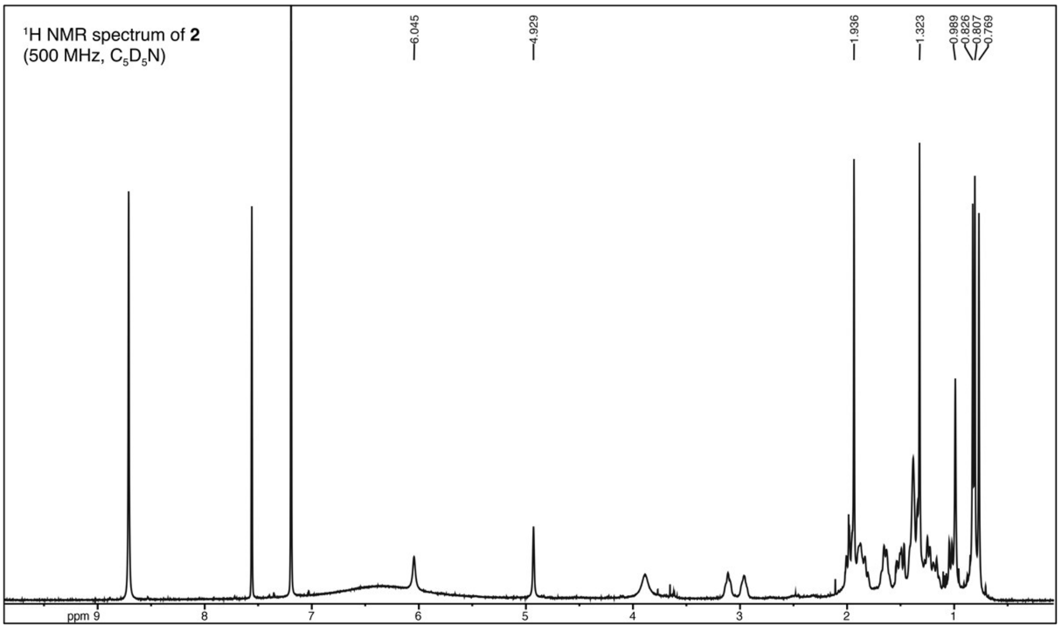

^{1}H NMR spectrum of **2**
(500 MHz, C_5D_5N)
6.045
4.929
1.936
1.323
0.989
0.826
0.807
0.769
ppm 9
8
7
6
5
4
3
2
1

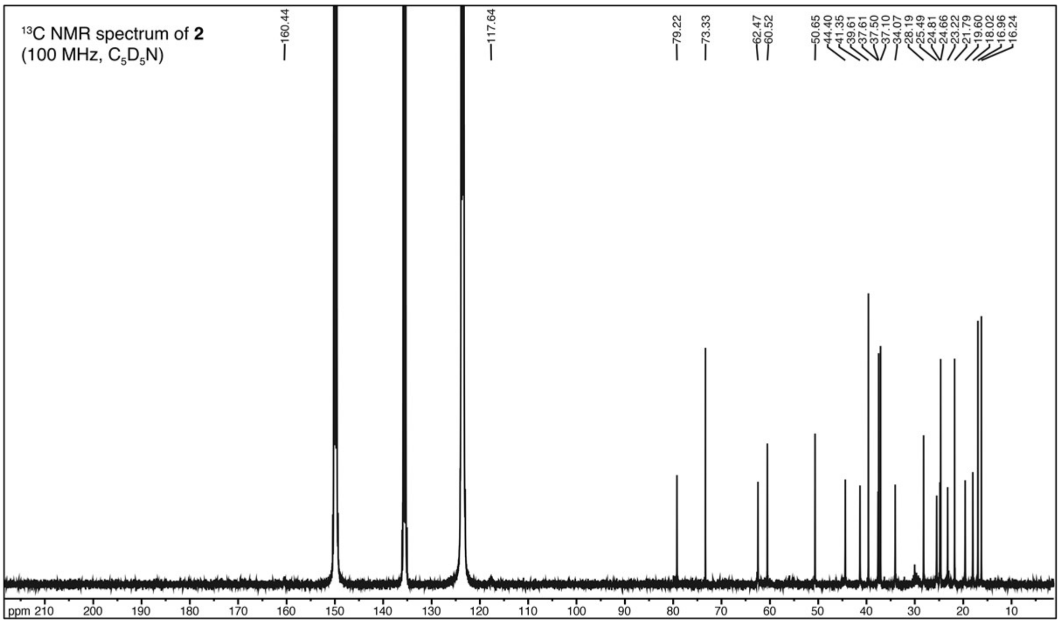

^{13}C NMR spectrum of **2**
(100 MHz, C_5D_5N)
160.44
117.64
79.22
73.33
62.47
60.52
50.65
44.40
41.35
39.61
37.61
37.50
37.10
34.07
28.19
25.49
24.81
24.66
23.22
21.79
19.60
18.02
16.96
16.24
ppm 210
200
190
180
170
160
150
140
130
120
110
100
90
80
70
60
50
40
30
20
10

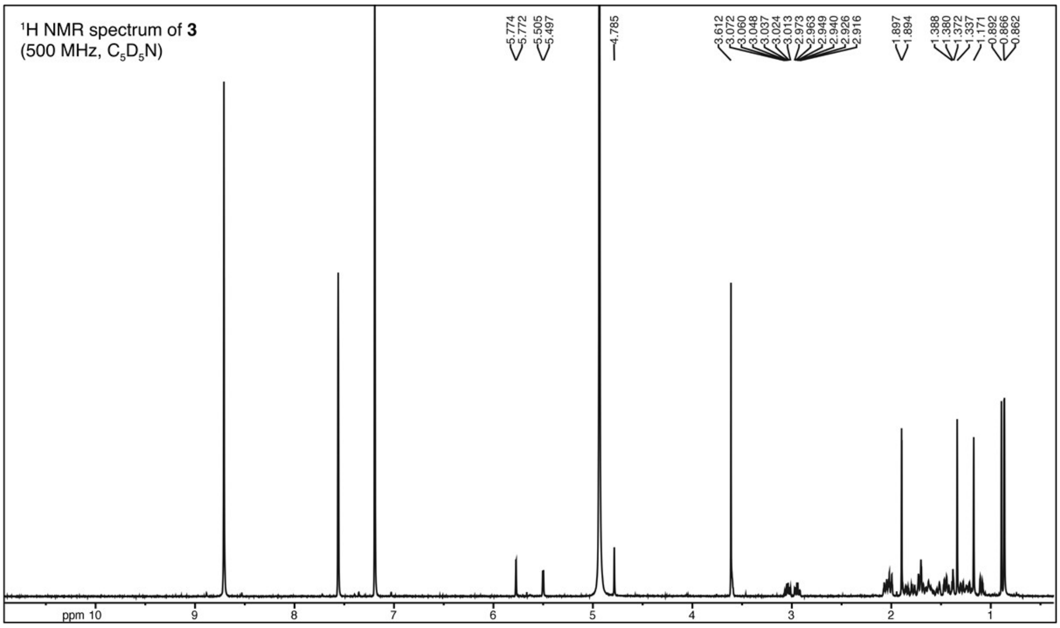

1H NMR spectrum of 3
(500 MHz, C_5D_5N)
5.774
5.772
5.505
5.497
4.785
3.612
3.072
3.060
3.048
3.037
3.024
3.013
2.973
2.963
2.949
2.940
2.926
2.916
1.897
1.894
1.388
1.380
1.372
1.337
1.171
0.892
0.866
0.862
ppm 10
9
8
7
6
5
4
3
2
1

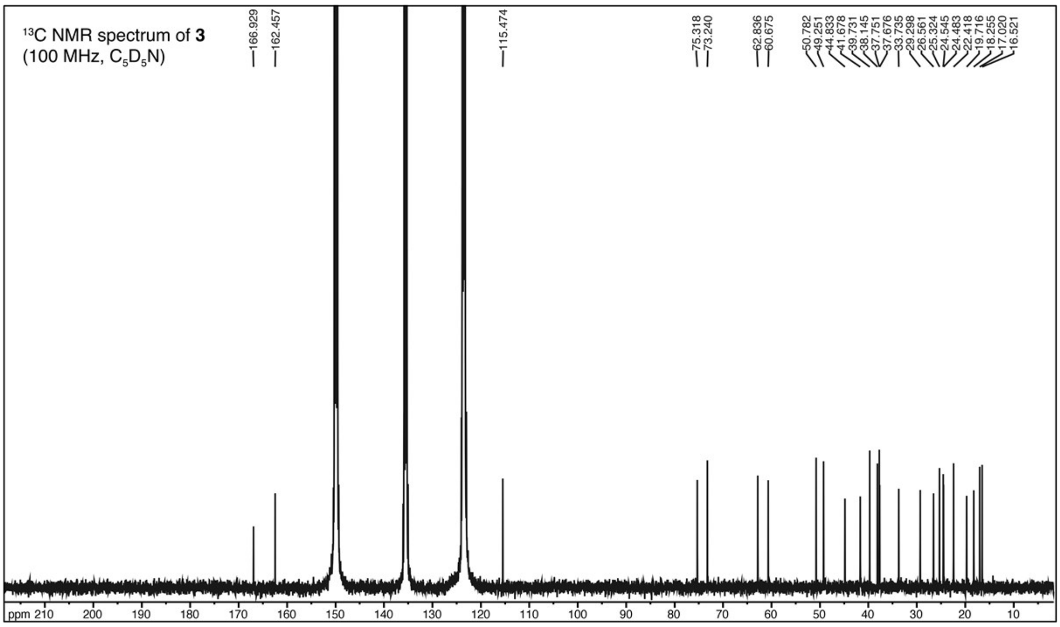

13C NMR spectrum of 3
(100 MHz, C_5D_5N)
166.929
162.457
115.474
75.318
73.240
62.836
60.675
50.782
49.251
44.833
41.678
39.731
38.145
37.751
37.676
33.735
29.298
26.561
25.324
24.545
24.483
22.418
19.716
18.255
17.020
16.521
ppm 210
200
190
180
170
160
150
140
130
120
110
100
90
80
70
60
50
40
30
20
10

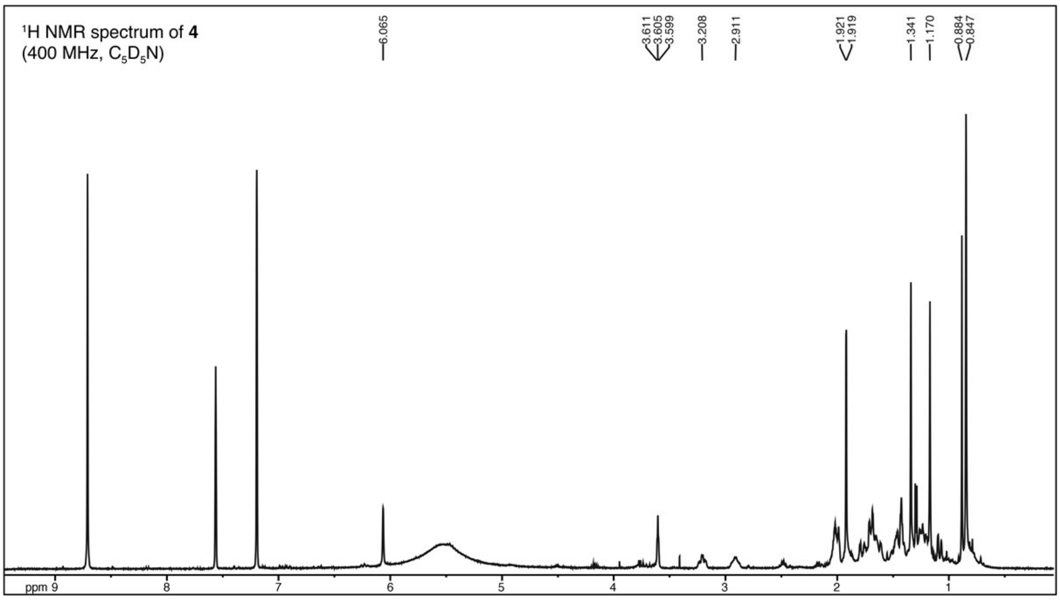

^{1}H NMR spectrum of **4**
(400 MHz, C_5D_5N)
6.065
3.611
3.605
3.599
3.208
2.911
1.921
1.919
1.341
1.170
0.884
0.847
ppm 9
8
7
6
5
4
3
2
1

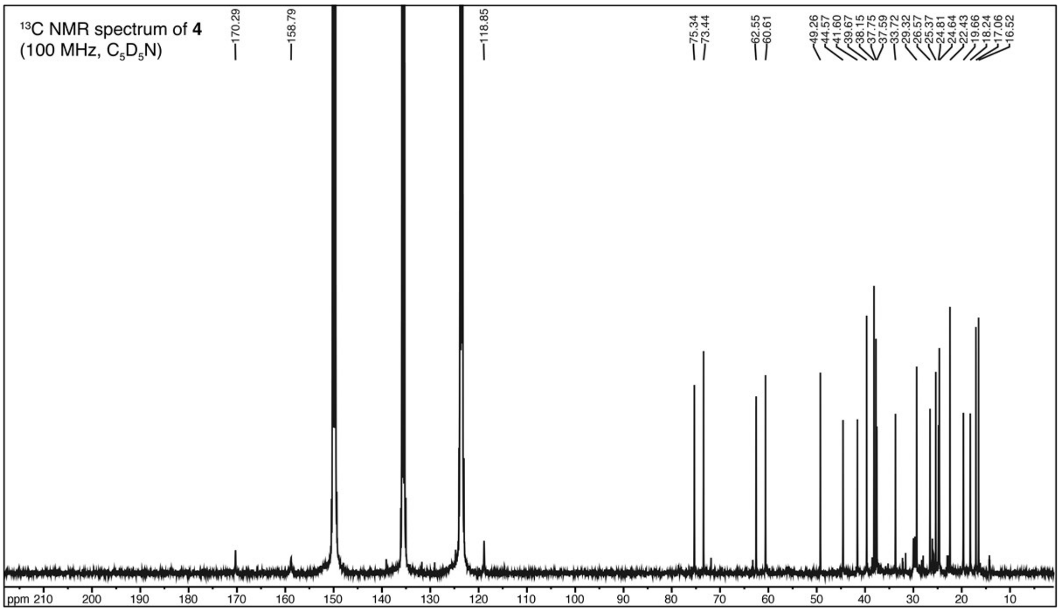

^{13}C NMR spectrum of **4**
(100 MHz, C_5D_5N)
170.29
158.79
118.85
75.34
73.44
62.55
60.61
49.26
44.57
41.60
39.67
38.15
37.75
37.59
33.72
29.32
26.57
25.37
24.81
24.64
22.43
19.66
18.24
17.06
16.52
ppm 210
200
190
180
170
160
150
140
130
120
110
100
90
80
70
60
50
40
30
20
10

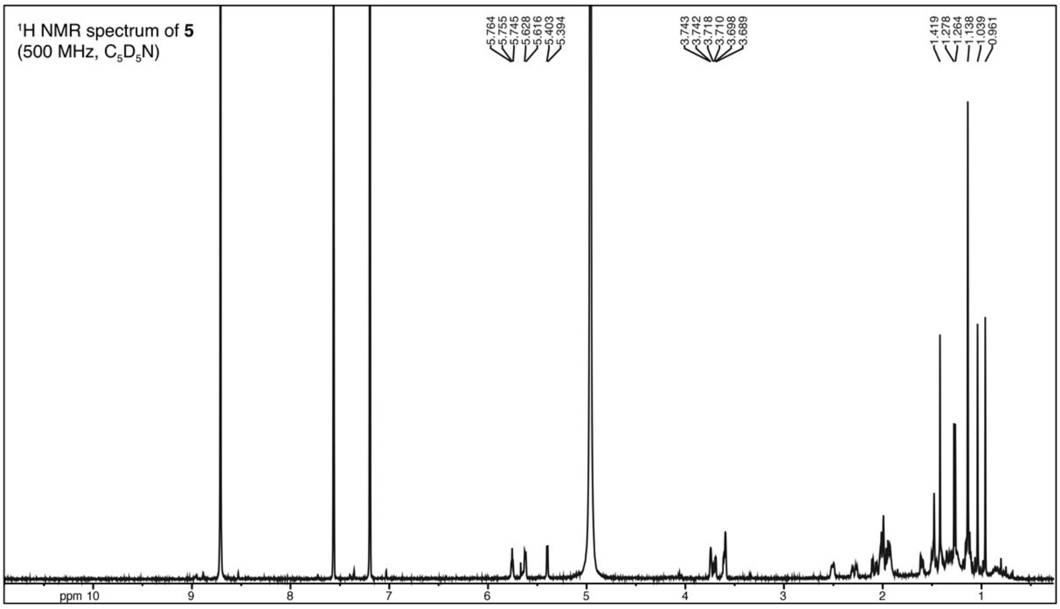

^{1}H NMR spectrum of **5**
(500 MHz, C_5D_5N)
5.764
5.755
5.745
5.628
5.616
5.403
5.394
3.743
3.742
3.718
3.710
3.698
3.689
1.419
1.278
1.264
1.138
1.039
0.961
ppm 10
9
8
7
6
5
4
3
2
1

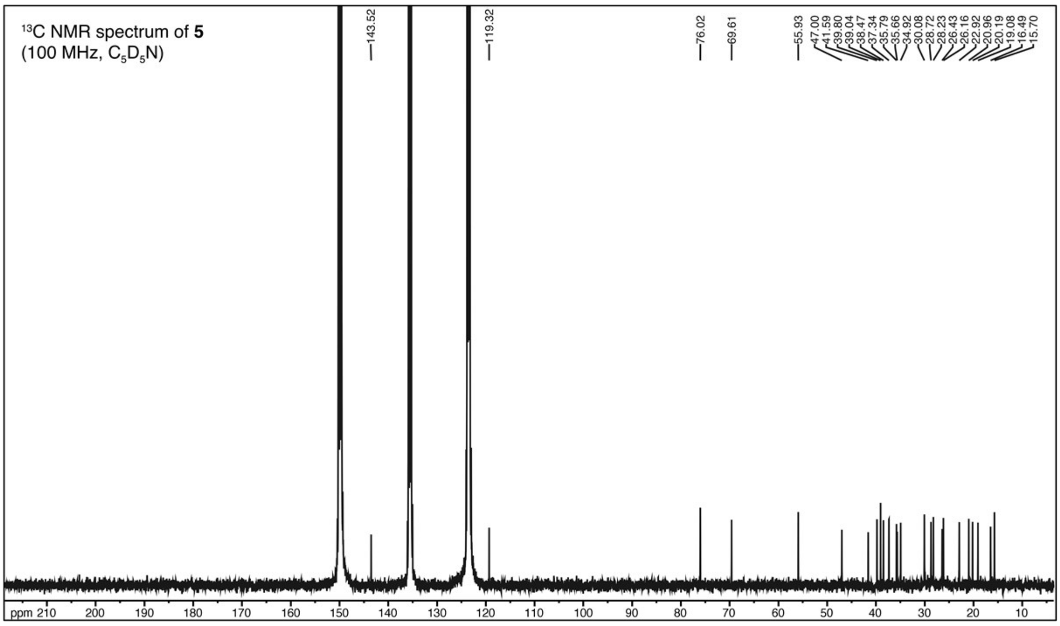

^{13}C NMR spectrum of **5**
(100 MHz, C_5D_5N)
143.52
119.32
76.02
69.61
55.93
47.00
41.59
39.80
39.04
38.47
37.34
35.79
35.66
34.92
30.08
28.72
28.23
26.43
26.16
22.92
20.96
20.19
19.08
16.49
15.70
ppm 210
200
190
180
170
160
150
140
130
120
110
100
90
80
70
60
50
40
30
20
10

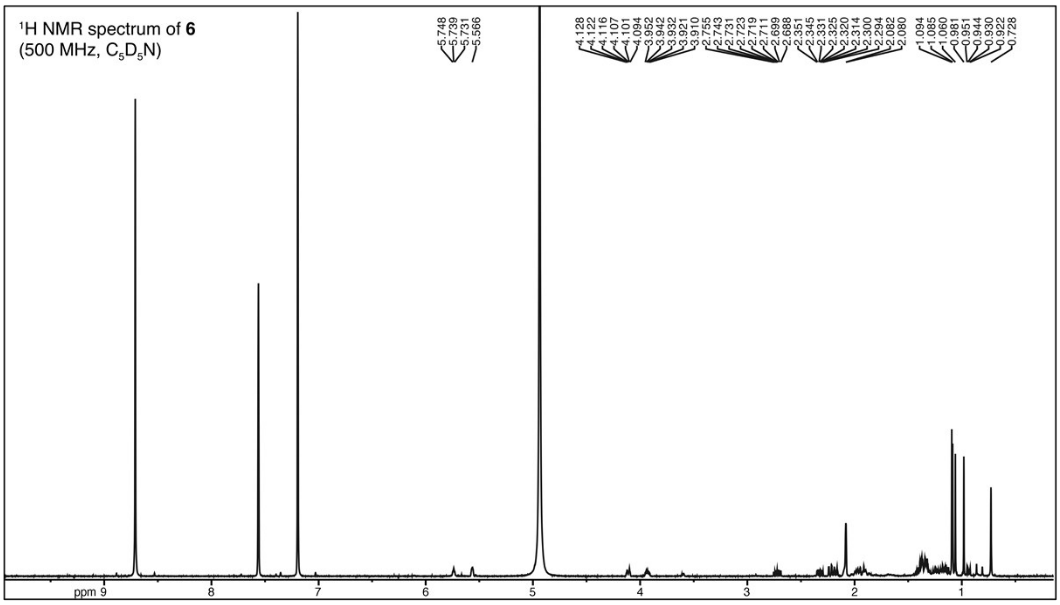
1H NMR spectrum of 6
(500 MHz, C_5D_5N)
5.748 5.739 5.731 5.566
4.128 4.122 4.116 4.107 4.101 4.094 3.952 3.942 3.932 3.921 3.910 2.755 2.743 2.731 2.723 2.719 2.711 2.699 2.688 2.351 2.345 2.331 2.325 2.320 2.314 2.300 2.294 2.082 2.080
1.094 1.085 1.060 0.981 0.951 0.944 0.930 0.922 0.728
ppm 9 8 7 6 5 4 3 2 1

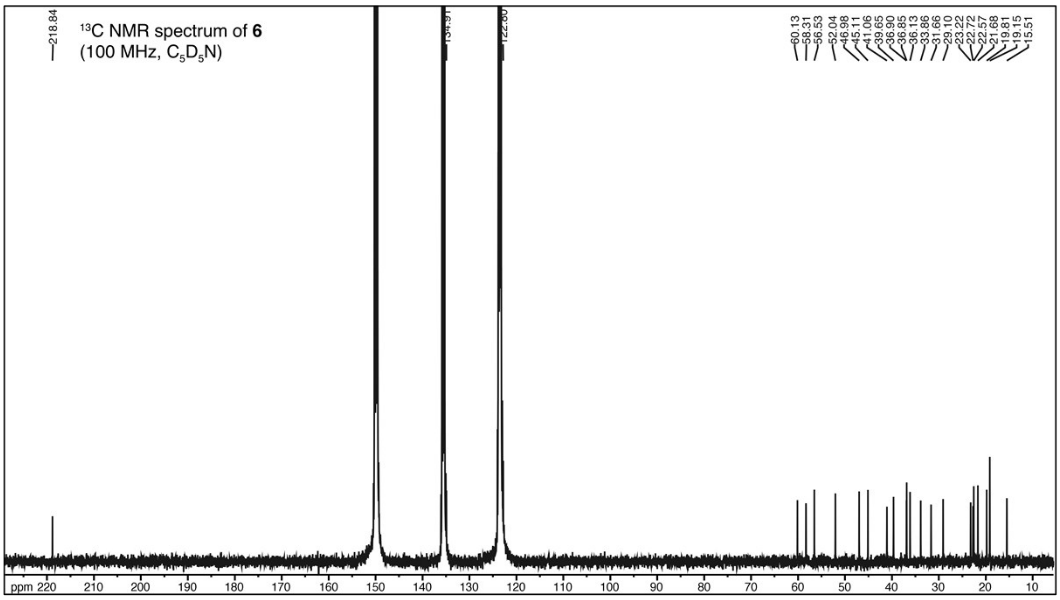
218.84
13C NMR spectrum of 6
(100 MHz, C_5D_5N)
60.13 58.31 56.53 52.04 46.98 45.11 41.06 39.65 36.90 36.85 36.13 33.86 31.66 29.10 23.22 22.72 22.57 21.68 19.81 19.15 15.51
ppm 220 210 200 190 180 170 160 150 140 130 120 110 100 90 80 70 60 50 40 30 20 10

Computational Methodology

Quantum mechanical calculations were performed using the Gaussian 09 software.[8] Computations were carried out to investigate the conformational preferences of C3-*epi*-**5**, to predict the NMR shifts of diastereomers of **5**, to predict the specific rotation of **5**, and to examine carbocation reactivities in the proposed biosynthetic routes to **5** and **6**. For each of these applications, combinations of functionals and basis sets were selected on the basis of literature precedent. Specific details of methodology for each application are provided below, along with the computational data.

Preferred Conformation of the C3-□ Epimer of 5

During the assignment of the relative stereochemistry of **5**, the observation of NOESY cross-peaks between H-3 and both H_3-24 and H_3-25 was used as a diagnostic of the C-3 stereochemistry. If the C3 stereochemistry of **5** was □, then the observation of NOESY cross-peaks between H-3 and both H_3-24 and H_3-25 would require a significant fraction of molecules to have a boat-like conformation of the A ring. Density functional theory calculations were therefore performed to determine the conformational preferences of C3-*epi*-**5**. Geometry optimizations were conducted at the B3LYP[9]/6-31G(d,p) level of theory in pyridine, as modeled by the PCM[10] implicit solvent model. Different combinations of chair and boat-like conformations of the A and D rings were explored. The global minimum was found to have the A and D rings both in chair conformations. A conformer having an A-ring chair and a D-ring boat lay 1.2 kcal/mol higher in energy. Conformers in which the A ring adopted a boat-like conformation, with the D ring in either a chair or boat-like conformation, lay at least 3.8 kcal/mol higher than the global minimum. Therefore, A-ring boat conformers would not be expected to contribute significantly to the conformer distribution of C3-*epi*-**5** at room temperature (0.1%), and a NOESY cross-peak between H-3 and H_3-24 (which are *trans*-diaxial in the A-ring chair conformer) would not be expected to be observed.

*Optimized geometries of conformers of C3-*epi-***5** *at the PCM$_{pyridine}$/B3LYP/6-31G(d,p) level of theory*

Gibbs free energies (G) for these species are corrected to a standard state of 1 mol/L (25 °C).

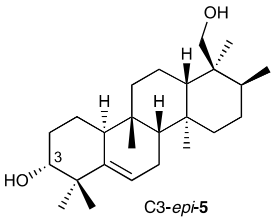

C3-*epi*-**5**

A ring chair, D ring chair (global minimum)

C	3.466387	0.133999	-0.984116
C	2.200096	0.415821	-0.106483
C	1.282493	-0.810365	0.239033
C	2.196204	-1.926893	0.819095
C	3.401180	-2.260129	-0.072779
C	4.301297	-1.041252	-0.362775
C	1.353213	1.615369	-0.564174
C	0.441552	2.081034	0.576317
C	-0.504699	0.994881	1.145670
C	0.305641	-0.326548	1.386541
C	-1.737294	0.783142	0.190294
C	-2.484124	-0.531627	0.420742
C	-1.985172	-1.473415	1.233280
C	-0.650452	-1.412448	1.926139
C	-3.768892	-0.774845	-0.403625
C	-4.665467	0.492124	-0.358391
C	-3.910440	1.770260	-0.713768
C	-2.731945	1.970821	0.238850
O	-5.780701	0.284078	-1.233541
C	-3.366920	-1.097519	-1.867256
C	-4.603913	-1.957053	0.126681
C	-0.992624	1.493387	2.530979
C	0.521128	-1.399544	-0.970097
C	5.130178	-0.683089	0.886625
C	4.332577	1.417155	-1.085030
O	4.433111	2.105207	0.161034
C	3.155942	-0.208122	-2.463026
H	-1.353787	0.765057	-0.839444
H	-2.216796	2.906711	0.000769
H	-3.129414	2.080949	1.253758
H	-3.572794	1.723304	-1.755997
H	-4.597244	2.624477	-0.644053
H	-5.029061	0.591009	0.679035
H	-6.358736	1.054254	-1.147914
H	-2.786766	-2.024625	-1.895211
H	-2.756570	-0.311608	-2.320082
H	-4.258906	-1.226160	-2.485717
H	-4.080566	-2.910092	0.007279
H	-5.540586	-2.015812	-0.431823
H	-4.845579	-1.831348	1.187550
H	-2.544344	-2.390460	1.397768
H	-0.185636	-2.402805	1.864995
H	-0.808908	-1.253229	3.002937
H	0.992983	-0.081574	2.208243
H	-0.169236	1.470209	3.252867
H	-1.808705	0.883240	2.928477
H	-1.346628	2.527146	2.479362
H	1.185009	-1.953274	-1.635202
H	0.022419	-0.641504	-1.576241

```
H   -0.245905   -2.098966   -0.630610
H    2.620409    0.720782    0.860613
H    0.763758    1.370132   -1.455562
H    1.998249    2.452765   -0.844907
H   -0.139766    2.956531    0.264072
H    1.086679    2.428736    1.395238
H    1.618671   -2.844491    0.979356
H    2.554044   -1.615594    1.809158
H    3.053433   -2.701227   -1.013488
H    4.001290   -3.040800    0.411715
H    5.024232   -1.342888   -1.135393
H    5.700833   -1.562958    1.206064
H    4.507508   -0.370369    1.729693
H    5.835129    0.127110    0.693502
H    3.899200    2.083409   -1.844752
H    5.329695    1.125054   -1.450765
H    4.976297    2.890414    0.012777
H    2.790065   -1.225575   -2.598251
H    4.068431   -0.114423   -3.064818
H    2.412634    0.473341   -2.887301
0 imaginary frequencies
E = -1128.442515
G = -1127.858939
```

A ring chair, D ring boat (G_{rel} = 1.2 kcal/mol)

```
C    3.366294    0.160165   -0.953439
C    2.133787    0.550699   -0.050152
C    1.265789   -0.629730    0.502293
C    2.198598   -1.595928    1.318670
C    3.698422   -1.471829    0.994311
C    3.965117   -1.221683   -0.497672
C    1.246778    1.649894   -0.662164
C    0.303359    2.241138    0.388788
C   -0.634710    1.209749    1.058560
C    0.218627   -0.018522    1.516908
C   -1.801529    0.806525    0.087794
C   -2.503622   -0.497839    0.472712
C   -2.002224   -1.292786    1.428871
C   -0.708831   -1.068080    2.165969
C   -3.734214   -0.919079   -0.361454
C   -4.677540    0.299286   -0.560710
C   -3.949688    1.543111   -1.066346
C   -2.842331    1.939231   -0.090584
C   -3.242418   -1.448439   -1.734838
C   -4.554879   -2.038134    0.309999
O   -5.729412   -0.088976   -1.452153
C   -1.212473    1.872591    2.336179
C    0.594978   -1.457831   -0.617943
C    5.452929   -1.415937   -0.830953
C    4.413573    1.292512   -0.838852
O    4.895958    1.421754    0.500744
C    3.019497    0.077321   -2.458374
H   -1.361543    0.657458   -0.908104
H   -2.350800    2.853620   -0.437597
H   -3.304678    2.177375    0.873790
H   -3.542754    1.353279   -2.066943
H   -4.671049    2.364690   -1.170341
H   -5.106699    0.537621    0.427869
H   -6.346638    0.653039   -1.505390
H   -2.633282   -2.345174   -1.587732
H   -2.630822   -0.718274   -2.271430
H   -4.093175   -1.705296   -2.370996
H   -3.995832   -2.976747    0.364026
```

H	-5.461259	-2.221186	-0.271430
H	-4.851125	-1.759176	1.326948
H	-2.528489	-2.204780	1.697791
H	-0.192228	-2.030294	2.262963
H	-0.930734	-0.770019	3.201165
H	0.856811	0.379461	2.317848
H	-0.428557	1.978927	3.093957
H	-2.028023	1.289316	2.773023
H	-1.595750	2.875801	2.127485
H	1.338049	-2.015724	-1.192373
H	0.023683	-0.849233	-1.321804
H	-0.092179	-2.192838	-0.191656
H	2.587660	1.001316	0.842968
H	0.677027	1.261078	-1.514087
H	1.865746	2.459670	-1.065113
H	-0.283490	3.058733	-0.046185
H	0.917097	2.700122	1.176800
H	1.880771	-2.630035	1.138460
H	2.065882	-1.426704	2.393590
H	4.207673	-2.391437	1.310066
H	4.149483	-0.657154	1.569042
H	3.422211	-1.996834	-1.056442
H	5.754852	-2.442537	-0.595798
H	6.081705	-0.738639	-0.245529
H	5.667631	-1.250251	-1.892180
H	3.959103	2.237525	-1.168387
H	5.245485	1.084784	-1.525711
H	5.498591	2.176658	0.520858
H	2.200158	-0.618068	-2.648571
H	3.886470	-0.270889	-3.030347
H	2.730918	1.050137	-2.868681

0 imaginary frequencies
E = -1128.441905
G = -1127.856975

A ring boat, D ring chair (G_{rel} = 5.8 kcal/mol)

C	-3.542351	0.168162	0.912858
C	-2.224408	0.418338	0.106676
C	-1.325206	-0.831084	-0.198949
C	-2.239521	-1.913572	-0.841270
C	-3.501637	-2.217319	-0.021432
C	-4.378637	-0.974376	0.234866
C	-1.369878	1.590453	0.618043
C	-0.383071	2.034469	-0.466421
C	0.564977	0.926305	-0.986415
C	-0.270217	-0.369053	-1.287411
C	1.715019	0.650348	0.045605
C	2.498494	-0.627342	-0.261272
C	2.004305	-1.561358	-1.084362
C	0.671806	-1.492237	-1.780360
C	3.839229	-0.785115	0.468671
C	4.794222	0.396838	0.045108
C	4.036390	1.639859	-0.456572
C	2.691318	1.855520	0.242030
O	5.748950	0.719190	1.065283
C	3.576067	-0.778784	1.999003
C	4.565769	-2.103503	0.148566
C	1.161013	1.421124	-2.327401
C	-0.647710	-1.454066	1.046683
C	-5.128810	-0.578617	-1.052497
C	-4.374013	1.475644	0.984412
O	-4.390813	2.178959	-0.257312
C	-3.317602	-0.199805	2.400415

H	1.229156	0.497315	1.015179
H	2.861100	2.022756	1.312455
H	2.238911	2.782069	-0.120957
H	4.689915	2.510706	-0.331196
H	3.865902	1.531953	-1.533136
H	5.417937	0.030906	-0.777384
H	5.273414	1.144566	1.792133
H	2.908873	-1.606983	2.257022
H	3.105771	0.140799	2.357631
H	4.511989	-0.914417	2.547076
H	3.999660	-2.975791	0.490580
H	5.535889	-2.109787	0.655782
H	4.750217	-2.214763	-0.924684
H	2.581284	-2.460418	-1.285725
H	0.184065	-2.469690	-1.695942
H	0.847491	-1.370976	-2.859648
H	-0.901558	-0.095613	-2.144408
H	0.371284	1.516131	-3.080957
H	1.920630	0.740081	-2.719630
H	1.621903	2.407763	-2.218000
H	-1.335295	-2.091011	1.605973
H	-0.268885	-0.708306	1.748190
H	0.195126	-2.082977	0.750732
H	-2.583253	0.740200	-0.879629
H	-0.839527	1.324550	1.540134
H	-2.005079	2.445229	0.866992
H	0.200213	2.894355	-0.116638
H	-0.971089	2.402211	-1.318671
H	-1.683677	-2.847835	-0.979873
H	-2.532394	-1.581430	-1.845724
H	-3.221144	-2.679466	0.931500
H	-4.097480	-2.973587	-0.548103
H	-5.150672	-1.261413	0.964360
H	-5.712207	-1.435864	-1.408263
H	-4.452325	-0.283271	-1.859549
H	-5.813867	0.254912	-0.889505
H	-3.960772	2.121309	1.772507
H	-5.396469	1.209537	1.295815
H	-4.921215	2.976248	-0.128489
H	-2.970284	-1.223115	2.539493
H	-4.260605	-0.103842	2.952713
H	-2.591306	0.466799	2.874632

0 imaginary frequencies
E = -1128.434474
G = -1127.849748

A ring boat, D ring boat (G_{rel} = 3.8 kcal/mol)

C	-3.297824	0.276039	0.850394
C	-2.050064	0.545815	-0.078085
C	-1.174803	-0.696443	-0.450867
C	-2.096072	-1.760668	-1.151268
C	-3.600599	-1.603307	-0.863174
C	-3.887045	-1.156802	0.577522
C	-1.173603	1.717126	0.397759
C	-0.207065	2.161618	-0.703045
C	0.745136	1.049970	-1.204121
C	-0.103503	-0.229091	-1.517425
C	1.871878	0.764226	-0.156623
C	2.606825	-0.551548	-0.398909
C	2.118660	-1.477589	-1.234188
C	0.824119	-1.368808	-1.995060
C	3.941894	-0.769764	0.351040
C	4.028563	0.198788	1.554290

C	3.880641	1.677885	1.149340
C	2.907700	1.906952	-0.027416
C	4.076847	-2.214917	0.878240
C	5.140628	-0.497566	-0.592818
O	3.004722	-0.187304	2.486692
C	1.372220	1.538658	-2.533159
C	-0.531628	-1.372453	0.783288
C	-5.378423	-1.309629	0.916145
C	-4.344490	1.378924	0.566404
O	-4.804185	1.324539	-0.786292
C	-2.974910	0.400084	2.357782
H	1.401482	0.681568	0.829424
H	2.403579	2.869172	0.102723
H	3.476180	1.986917	-0.959189
H	3.545626	2.230796	2.036163
H	4.866268	2.086057	0.899537
H	5.011959	0.056917	2.029695
H	3.059612	0.416804	3.239393
H	4.130064	-2.936662	0.058587
H	3.236774	-2.480721	1.521673
H	4.999852	-2.315963	1.461422
H	5.129931	-1.210878	-1.422148
H	6.090492	-0.616613	-0.057816
H	5.119867	0.506896	-1.022580
H	2.676464	-2.398225	-1.397526
H	0.302569	-2.332048	-1.945913
H	1.051028	-1.233493	-3.063211
H	-0.721353	0.059258	-2.379429
H	0.612663	1.578968	-3.321862
H	2.180074	0.884799	-2.874371
H	1.783683	2.547293	-2.428547
H	-1.288761	-1.865760	1.396732
H	0.012982	-0.679769	1.427647
H	0.176137	-2.144001	0.470541
H	-2.486400	0.871472	-1.031934
H	-0.619542	1.445431	1.303304
H	-1.798049	2.573746	0.676714
H	0.371735	3.030825	-0.369102
H	-0.800832	2.509481	-1.560254
H	-1.777272	-2.762837	-0.840013
H	-1.951192	-1.726157	-2.237369
H	-4.102152	-2.560052	-1.058601
H	-4.048635	-0.874980	-1.546050
H	-3.350217	-1.847127	1.242867
H	-5.673936	-2.360756	0.825609
H	-6.002128	-0.725524	0.233029
H	-5.605933	-0.995218	1.940221
H	-3.896400	2.361094	0.773576
H	-5.187896	1.262118	1.260752
H	-5.400559	2.072636	-0.920986
H	-2.158848	-0.261343	2.654409
H	-3.851039	0.132428	2.958694
H	-2.692668	1.420577	2.634713

0 imaginary frequencies
E = -1128.436962
G = -1127.852820

General Notes on NMR Shift Calculations

NMR chemical shifts were computed by means of $PCM_{pyridine}$/mPW1PW91[11]/6-311+G(2d,p)[12] single-point calculations on $PCM_{pyridine}$/B3LYP/6-31G(d,p) geometries to best match NMR experiments (which were performed in pyridine-d_5). The computed chemical shifts were scaled using the training set in accordance with Tantillo's method.[13] The scaling factors are shown below.

Table S2. Scaling factors for the present level of theory.

$$\delta = \frac{b - \sigma}{-m}$$

	^{1}H	^{13}C
m	-1.0858	-1.0582
b	31.8360	185.4211

Table S3. Comparison of Computed NMR shifts for Each Diastereomer of 5.

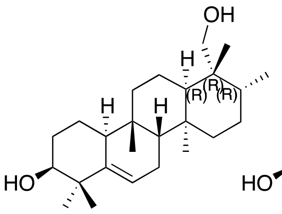
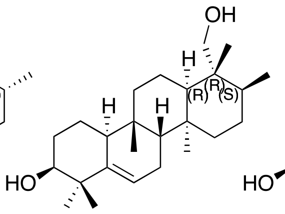
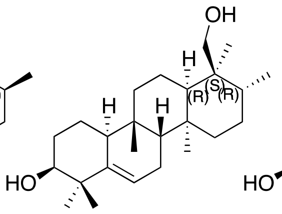
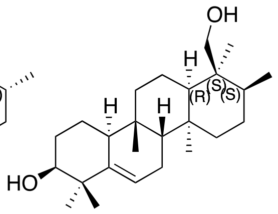

(13R,17R*,18R*)*-**5** *(13R*,17S*,18R*)*-**5** *(13R*,17R*,18S*)*-**5** *(13R*,17S*,18S*)*-**5**

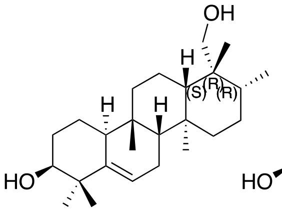
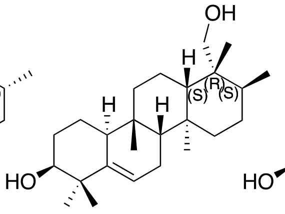
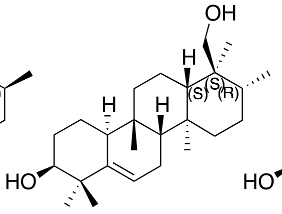
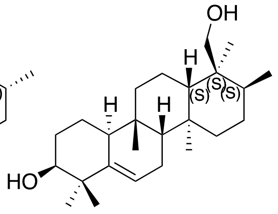

(13S,17R*,18R*)*-**5** *(13S*,17S*,18R*)*-**5** *(13S*,17R*,18S*)*-**5** *(13S*,17S*,18S*)*-**5**

	**^{1}H**			**^{13}C**			**Probability[c]**
**Compound**	**CMAD[a]**	**Largest deviation (ppm)**	**$R^{2,b}$**	**CMAD[a]**	**Largest deviation (ppm)**	**$R^{2,b}$**	**(%)**
13*R**,17*R**,18*R**	0.31	0.97	0.881	3.9	11.0	0.973	0.00
13*R**,17*S**,18*R**	0.34	0.94	0.861	3.6	14.8	0.971	0.00
13*R**,17*R**,18*S**	0.32	1.13	0.876	3.3	10.3	0.975	0.00
13*R**,17*S**,18*S**	0.35	0.91	0.851	3.7	14.9	0.969	0.00
13*S**,17*R**,18*R**[d]	0.24	0.70	0.946	2.7	10.0	0.986	0.00
13*S**,17*S**,18*R**[d]	0.19	0.66	0.969	2.1	5.1	0.994	0.02
13*S**,17*R**,18*S**[d]	0.27	0.69	0.929	2.2	8.9	0.992	0.00
**13*S**,17*S**,18*S**[d]**	**0.21**	**0.52**	**0.972**	**1.8**	**5.8**	**0.996**	**99.8**

[a]CMAD = Computed Mean Absolute Deviation; $(1/n)\Sigma|\delta_{comp}-\delta_{exp}|$, where δ_{comp} refers to the scaled computed chemical shifts.
[b]R^2 = linear regression coefficient between δ_{comp} and δ_{exp}.
[c]Computed with Goodman's DP4 function.[14]
[d]Weighted average of conformations < 2.0 kcal/mol.

The gross conformational change upon inversion of stereochemistry of C-13 was captured in the NMR calculations, resulting in poor fit for all 13R* diastereomers (i.e. those corresponding to a *cis* C/D ring junction). Our efforts turned to further distinguish between the 13S* diastereomers by computing the weighted averages of the lowest energy conformers (<2 kcal/mol). Below is an example. Contribution percentages were calculated with the following equations:

$$K_{eq,i} = e^{-\frac{\Delta G_i}{RT}}, for\ i\ conformer$$

$$\%\ contribution\ of\ i = \frac{K_{eq,i}}{\Sigma(K_{eq,n})} \times 100$$

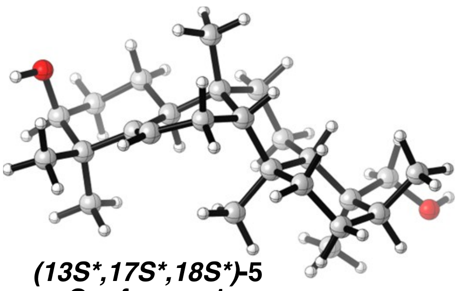

***(13S*,17S*,18S*)*-5**
Conformer 1
ΔΔG = 0.0 (72.1%)

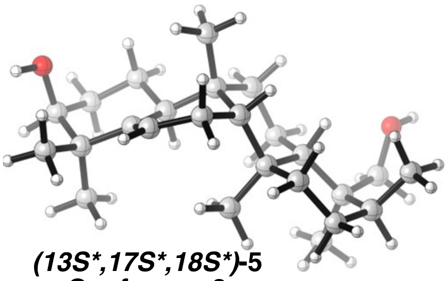

***(13S*,17S*,18S*)*-5**
Conformer 2
ΔΔG = 1.1 (19.5%)

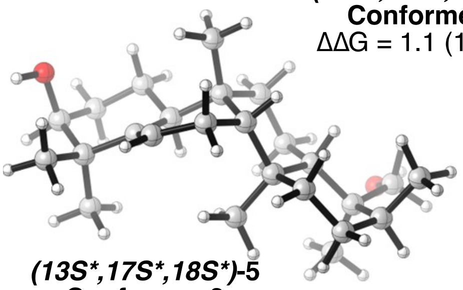

***(13S*,17S*,18S*)*-5**
Conformer 3
ΔΔG = 1.6 (8.4%)

Table S4. Comparison of Weighted Average Computed NMR shifts for (13*S,17*S**,18*S**)-5.**

	^{1}H			^{13}C			ΔE
**Compound**	**CMAD [a]**	**Largest deviation (ppm)**	**R^2**	**CMAD [a]**	**Largest deviation (ppm)**	**R^2**	**(kcal/mol)**
SSS-1	0.21	0.45	0.970	1.8	5.8	0.996	0.0
SSS-2	0.19	0.40	0.957	1.8	6.0	0.995	1.1
SSS-3	0.22	0.43	0.935	2.1	1.8	0.994	1.6
***SSS*-Avg**	**0.21**	**0.44**	**0.972**	**1.8**	**5.8**	**0.996**	–

Table S5. Computed Chemical Shifts for (13*S,17*S**,18*S**)-5[a]**

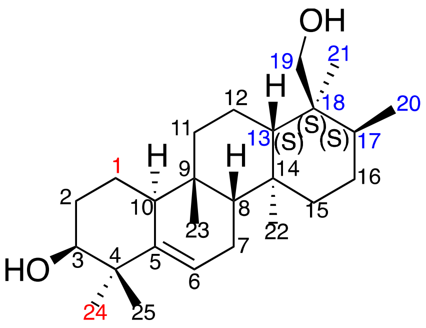

(13S,17S*,18S*)*-**5**

	^{1}H [δ, mult, *J* (Hz)]			^{13}C		
**Position**	**Experimental**	**Computed**[b]	**Abs. Diff.**	**Experimental**	**Computed**[b]	**Abs. Diff.**
1a	2.00 m[c]	1.56	0.44	21.0	21.4	0.4
1b	1.60 m	1.48	0.12			
2a	1.91-2.04 m[c]	1.77	ambiguous	30.1	27.4	2.7
2b	1.91-2.04 m[c]	1.69	ambiguous			
3	3.74 m	3.45	0.29	76.0	75.3	0.7
4				41.6	43.2	1.6
5				143.5	143.0	0.5
6	5.62 m	5.55	0.17	119.3	119.8	0.5
7a	2.29 m	2.20	0.09	22.9	22.7	0.2
7b	2.08 dd (19.0, 5.8)	2.01	0.07			
8	1.13 m[c]	1.08	0.05	55.9	54.1	1.8
9				35.8	39.6	3.8
10	2.51 m	2.36	0.15	37.3	37.1	0.2
11a	2.01 m[c]	1.92	0.09	38.5	36.8	1.7
11b	1.13 m[c]	1.05	0.08			
12a	1.38-1.49 m[c]	1.34	ambiguous	19.1	18.8	0.3
12b	1.38-1.49 m[c]	1.31	ambiguous			
13	1.48 m[c]	1.28	0.20	47.0	47.2	0.2
14				39.0	39.6	0.6
15a	1.49 m[c]	1.55	0.06	34.9	32.7	2.2
15b	1.15 m[c]	1.04	0.11			

16a	1.99 m[c]	1.95	0.04	26.2	24.7	1.5
16b	1.34 m[c]	1.24	0.10			
17	1.93 m[c]	1.55	0.38	35.7	35.9	0.2
18				39.8	42.1	2.3
19a	3.70 dd (10.2, 4.4)	3.36	0.34	69.6	68.0	2.8
19b	3.60 m	3.28	0.32			
20	1.27 d (7.1)	0.97	0.34	15.7	12.9	2.8
21	1.14 s	1.00	0.14	20.2	16.9	3.3
22	1.04 s	0.89	0.15	16.5	13.1	3.3
23	0.96 s	0.87	0.09	28.7	26.3	2.4
24	1.14 s	1.03	0.11	28.2	22.4	5.8
25	1.42 s	1.00	0.42	26.4	25.0	1.4

[a]Colored values correlate with colored regions in the figure; blue is the region of interest and red are the regions with the largest deviation.
[b]Weighted average of three lowest energy conformers.
[c]Indicates overlapping signals.

Molecular geometries of diastereomers of compound **5** *at the PCM$_{pyridine}$/B3LYP/ 6-31G(d,p) level of theory.*

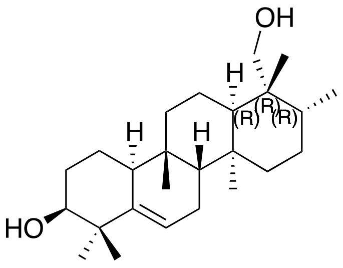

(13R,17R*,18R*)*-**5**

***(13R*,17R*,18R*)*-5**

C	-2.23102	-0.79481	0.51501
H	-2.49923	-1.02991	1.55462
C	-3.00213	-1.81896	-0.36402
H	-2.70691	-2.84494	-0.13183
H	-2.75514	-1.65191	-1.42085
C	-4.52163	-1.68864	-0.16040
H	-4.78377	-2.01503	0.85351
H	-5.05102	-2.35625	-0.84944
C	-5.03595	-0.25906	-0.39025
H	-6.10530	-0.20867	-0.14998
O	-4.95213	0.07437	-1.78179
H	-4.02181	0.28706	-1.95754
C	-4.27515	0.79520	0.48399
C	-4.59415	0.57990	1.98251
H	-5.66937	0.69691	2.15637
H	-4.07467	1.32965	2.58849
H	-4.30680	-0.40426	2.35662
C	-4.77220	2.20533	0.11642
H	-4.23124	2.97067	0.68193
H	-5.83570	2.29651	0.36216
H	-4.66396	2.41728	-0.94852
C	-2.78847	0.57528	0.17449
C	-2.04922	1.43338	-0.53919
H	-2.45723	2.40021	-0.82415
C	-0.63665	1.19168	-0.97959
H	0.00075	1.92111	-0.46209
H	-0.56912	1.47260	-2.03566
C	-0.06451	-0.24674	-0.79784
H	-0.39922	-0.84121	-1.66100
C	-0.66236	-0.90297	0.48955
C	-0.34626	-2.40909	0.67036
H	0.65440	-2.59027	1.06104
H	-0.44653	-2.97367	-0.26080
H	-1.04188	-2.84162	1.39884
C	1.51980	-0.18301	-0.90027
C	1.83386	0.64492	-2.18167
H	1.12461	0.38400	-2.97532
H	2.82484	0.42681	-2.57831
H	1.78164	1.72232	-2.01325
C	2.11880	0.47270	0.40292
H	1.85082	1.53421	0.38067
C	1.46809	-0.10967	1.68797
H	1.78157	0.49137	2.54497
H	1.85060	-1.11855	1.88356
C	-0.06858	-0.13487	1.69119
H	-0.44860	0.89344	1.69665
H	-0.41446	-0.59172	2.62857
C	2.14777	-1.58248	-1.13234
H	1.79983	-1.97338	-2.09832
H	1.81139	-2.29506	-0.38227
C	3.68204	-1.57787	-1.09443
H	4.10097	-1.04386	-1.95622
H	4.03996	-2.61094	-1.19181
C	4.24507	-0.98563	0.21062
H	3.86854	-1.61514	1.03010
C	5.77854	-1.11594	0.22577
H	6.22346	-0.74505	1.15328
H	6.24348	-0.57904	-0.60830
H	6.06179	-2.16959	0.12682
C	3.70184	0.45469	0.50371
C	4.34523	1.47808	-0.46537
H	4.27109	1.15635	-1.50777
H	5.41591	1.55194	-0.22993

O	3.72462	2.75639	-0.29513
H	4.17949	3.37710	-0.87912
C	4.14389	0.89296	1.92341
H	3.91621	0.13964	2.68156
H	3.65767	1.82953	2.21093
H	5.22317	1.06887	1.95170

0 Imaginary Frequencies

E = -1128.43857861

H = -1127.776515

G = -1127.856213

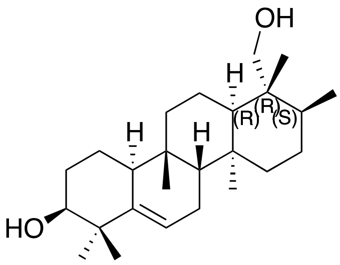

(13R,17S*,18R*)*-**5**

***(13R*,17S*,18R*)*-5**

C	1.82848	-0.77412	-0.00087
H	1.54802	-0.89812	-1.05534
C	2.77174	-1.96945	0.30986
H	2.25091	-2.91658	0.13528
H	3.04833	-1.96184	1.36896
C	4.06194	-1.92950	-0.51301
H	3.84673	-2.05937	-1.58003
H	4.71748	-2.75761	-0.22049
C	4.81124	-0.61132	-0.28700
H	5.71848	-0.59133	-0.90332
O	5.28603	-0.53535	1.06642
H	4.53712	-0.26072	1.61579
C	3.94236	0.62714	-0.64804
C	3.67194	0.63981	-2.17556
H	4.61867	0.62472	-2.72771
H	3.13580	1.55258	-2.45206
H	3.07648	-0.20966	-2.51614
C	4.74737	1.90035	-0.32428
H	4.21648	2.80080	-0.64655
H	5.70291	1.87243	-0.85900
H	4.96695	1.99024	0.74160
C	2.60126	0.54083	0.11064
C	2.09559	1.58292	0.78553
H	2.67737	2.49615	0.87708
C	0.72086	1.63706	1.39522
H	0.32033	2.64328	1.23788
H	0.80393	1.54975	2.48805
C	-0.25704	0.55620	0.87558
H	-1.01682	0.43195	1.65651
C	0.50049	-0.81904	0.84243
C	0.81664	-1.20266	2.31191
H	-0.07175	-1.06510	2.93742
H	1.62387	-0.60085	2.73940
H	1.10835	-2.25335	2.39421
C	-1.08422	0.96868	-0.40618
C	-0.18017	1.36269	-1.60130
H	-0.80360	1.68454	-2.44318
H	0.45442	0.55171	-1.95869
H	0.47352	2.19832	-1.33817
C	-2.03844	-0.19913	-0.88597
H	-2.27782	0.07867	-1.92334
C	-1.22486	-1.51685	-0.97952
H	-0.52447	-1.42520	-1.81802
H	-1.87681	-2.35399	-1.24947
C	-0.44421	-1.90613	0.28329
H	0.12833	-2.81794	0.07466
H	-1.14358	-2.17774	1.07678
C	-1.93255	2.23292	-0.10644
H	-2.27248	2.64248	-1.06769
H	-1.30726	3.01493	0.33502
C	-3.16896	1.98386	0.74655
H	-3.67770	2.93723	0.93890
H	-2.90291	1.58097	1.73272
C	-4.13300	1.03744	0.02366

H	-4.29930	1.47462	-0.97378
C	-5.49732	1.02653	0.73506
H	-5.92153	2.03716	0.71944
H	-5.40215	0.73531	1.78700
H	-6.20187	0.34492	0.25772
C	-3.48620	-0.36924	-0.23064
C	-4.31072	-1.10565	-1.32512
H	-3.73442	-1.95561	-1.71193
H	-4.47235	-0.41350	-2.16630
O	-5.56183	-1.57980	-0.82129
H	-5.98867	-2.07151	-1.53508
C	-3.52034	-1.22344	1.05306
H	-3.16148	-2.23825	0.86584
H	-4.54505	-1.31912	1.41441
H	-2.91760	-0.79355	1.85743

0 Imaginary Frequencies

E = -1128.43651072

H = -1127.774701

G = -1127.854882

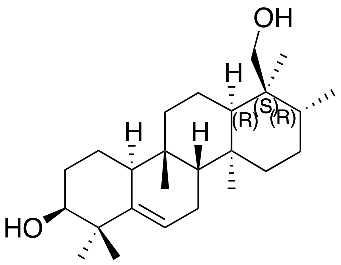

(13R,17R*,18S*)*-**5**

(13R*,17R*,18S*)-5

C	2.20216	-0.40208	-0.83704
H	2.42863	-0.03585	-1.84836
C	2.99564	-1.72973	-0.68056
H	2.67992	-2.47206	-1.41752
H	2.79366	-2.16257	0.30799
C	4.50699	-1.49116	-0.84227
H	4.72354	-1.21389	-1.88120
H	5.05644	-2.41868	-0.64591
C	5.04618	-0.40664	0.10261
H	6.10568	-0.22232	-0.11498
O	5.02248	-0.87823	1.45616
H	4.10285	-0.80546	1.75712
C	4.26313	0.94340	-0.03231
C	4.51863	1.57615	-1.42084
H	4.20693	0.94515	-2.25453
H	5.58672	1.78471	-1.54662
H	3.98058	2.52616	-1.50489
C	4.79077	1.93755	1.01833
H	4.72105	1.54314	2.03323
H	4.24210	2.88368	0.97245
H	5.84655	2.15307	0.82234
C	2.78722	0.57385	0.16750
C	2.08458	0.90459	1.25784
H	2.51336	1.56955	2.00387
C	0.68590	0.44891	1.54988
H	0.03897	1.33633	1.52912
H	0.65894	0.11549	2.59268
C	0.08993	-0.66932	0.64236
H	0.44479	-1.63303	1.03709
C	0.63517	-0.52304	-0.81646
C	0.29608	-1.69658	-1.76976
H	0.96238	-1.66276	-2.63958
H	-0.71803	-1.64385	-2.16343
H	0.42006	-2.67398	-1.29511
C	-1.48978	-0.68466	0.81860
C	-1.75159	-0.68107	2.35212
H	-1.63979	0.30648	2.80493
H	-1.04773	-1.36009	2.84711
H	-2.74933	-1.03905	2.60068
C	-2.13157	0.55876	0.09231
H	-1.85272	1.44887	0.66928
C	-1.52765	0.77274	-1.32280
H	-1.86103	1.74438	-1.69349
H	-1.92608	0.02856	-2.02219
C	0.00764	0.76600	-1.39103

H 0.39963 1.63717 -0.85313

H 0.31632 0.89194 -2.43780

C -2.12201 -1.99761 0.28431

H -1.74380 -2.84157 0.87691

H -1.81692 -2.19053 -0.74168

C -3.65588 -1.99136 0.30606

H -4.04080 -1.99805 1.33298

H -4.01883 -2.92265 -0.14723

C -4.26003 -0.80414 -0.46568

H -3.91650 -0.89729 -1.50699

C -5.79428 -0.93079 -0.48862

H -6.07832 -1.90450 -0.90247

H -6.27656 -0.16551 -1.10286

H -6.21941 -0.86914 0.51878

C -3.71931 0.56932 0.05600

C -4.21459 1.68126 -0.90944

H -5.31048 1.72490 -0.87331

H -3.93868 1.45353 -1.94186

O -3.67192 2.97592 -0.63982

H -4.01664 3.26608 0.21506

C -4.31569 0.92605 1.44242

H -5.36407 1.22892 1.34617

H -3.77227 1.75901 1.90153

H -4.29114 0.10174 2.15380

0 Imaginary Frequencies

E = -1128.43987606

H = -1127.777869

G = -1127.857392

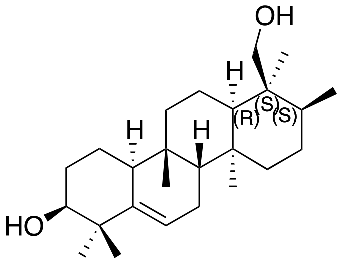

(13R,17S*,18S*)*-**5**

***(13R*,17S*,18S*)*-5**

C 1.75401 0.71965 0.14793

H 1.51607 0.65646 1.21867

C 2.68664 1.95366 0.00980

H 2.17560 2.85418 0.36501

H 2.92014 2.12856 -1.04523

C 4.01015 1.77874 0.75981

H 3.83997 1.72304 1.84135

H 4.65582 2.64710 0.58654

C 4.74507 0.52147 0.28055

H 5.67619 0.39847 0.84718

O 5.16405 0.68140 -1.08424

H 4.39197 0.50171 -1.64097

C 3.88733 -0.76375 0.45611

C 3.67725 -1.04445 1.96716

H 4.64467 -1.13068 2.47529

H 3.14176 -1.98949 2.09836

H 3.10427 -0.26653 2.47562

C 4.67454 -1.95812 -0.11698

H 4.15478 -2.90322 0.06528

H 5.65160 -2.01966 0.37427

H 4.84953 -1.85951 -1.19036

C 2.51815 -0.55172 -0.22308

C 1.98375 -1.46134 -1.05013

H 2.55784 -2.34340 -1.32120

C 0.59036 -1.40626 -1.61449

H 0.19059 -2.42491 -1.63187

H 0.64018 -1.11904 -2.67474

C -0.36771 -0.44093 -0.87701

H -1.14961 -0.18567 -1.60193

C 0.38954 0.91070 -0.61700

C 0.64312 1.56074 -2.00334

H -0.27161 1.53149 -2.60523

H 1.43160 1.05550 -2.56945

H 0.92841 2.61206 -1.90581

C -1.15795 -1.07689 0.33287

C -0.21433 -1.67614 1.40538

H	-0.80708	-2.11886	2.21386
H	0.45811	-0.94663	1.85604
H	0.40434	-2.47055	0.97903
C	-2.08862	-0.01037	1.04392
H	-2.32408	-0.47491	2.01227
C	-1.25793	1.25353	1.37238
H	-0.53002	0.99031	2.14911
H	-1.89486	2.02134	1.81936
C	-0.52424	1.87287	0.17657
H	0.06698	2.72695	0.53057
H	-1.26581	2.29869	-0.50174
C	-2.01971	-2.27384	-0.15595
H	-2.29978	-2.86357	0.72776
H	-1.41926	-2.94871	-0.77332
C	-3.31295	-1.89903	-0.87064
H	-3.83728	-2.81487	-1.17224
H	-3.11835	-1.34538	-1.79897
C	-4.21087	-1.08350	0.06721
H	-4.27188	-1.65308	1.00735
C	-5.64607	-1.00998	-0.48576
H	-6.07611	-2.01730	-0.50414
H	-5.67816	-0.63325	-1.51348
H	-6.30651	-0.38490	0.12123
C	-3.54561	0.28154	0.46338
C	-3.62139	1.24688	-0.74417
H	-4.59792	1.12840	-1.22834
H	-2.85934	1.00058	-1.49352
O	-3.48584	2.60857	-0.32002
H	-3.56871	3.16099	-1.10860
C	-4.38333	0.89319	1.61372
H	-5.38169	1.18033	1.27200
H	-3.92203	1.79183	2.02447
H	-4.50107	0.16649	2.42508

0 Imaginary Frequencies

E = -1128.43619626

H = -1127.774587

G = -1127.854445

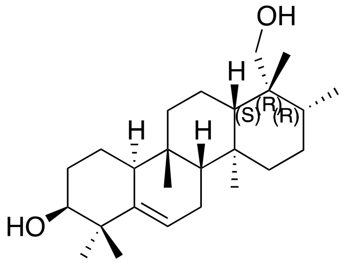

(13S,17R*,18R*)*-**5**

***(13S*,17R*,18R*)*-5 Conformer 1**

C	-1.78136	-0.71585	-0.23084
H	-1.23506	-0.95507	0.69156
C	-2.85216	-1.82783	-0.38533
H	-2.37021	-2.80969	-0.42508
H	-3.37984	-1.70641	-1.33666
C	-3.89589	-1.80909	0.73448
H	-3.43608	-2.05370	1.69903
H	-4.65966	-2.57237	0.54681
C	-4.58272	-0.44140	0.81477
H	-5.30726	-0.43955	1.63827
O	-5.36892	-0.20892	-0.36495
H	-4.76148	0.08814	-1.05814
C	-3.56801	0.71229	1.05107
C	-2.93348	0.55904	2.45880
H	-3.71504	0.53481	3.22707
H	-2.28184	1.41254	2.66848
H	-2.33505	-0.34793	2.56514
C	-4.33709	2.04780	1.04829
H	-5.13700	2.00813	1.79546
H	-4.79755	2.25773	0.08048
H	-3.68432	2.88505	1.31136
C	-2.45459	0.64338	-0.01699
C	-2.03119	1.73525	-0.67094
H	-2.54170	2.68251	-0.51942
C	-0.82683	1.79737	-1.57177
H	-0.29230	2.73059	-1.36294
H	-1.15846	1.90215	-2.61493

C	0.12063	0.58197	-1.45793
H	0.65783	0.51648	-2.41437
C	-0.73566	-0.72729	-1.40590
C	-1.46204	-0.85890	-2.76997
H	-0.75744	-0.68389	-3.59009
H	-2.28590	-0.14732	-2.87840
H	-1.87170	-1.86373	-2.90674
C	1.29282	0.75143	-0.40395
C	0.77443	1.09536	1.01068
H	-0.01698	1.84578	0.95958
H	1.56452	1.51363	1.63701
H	0.37403	0.22814	1.53868
C	2.16216	-0.55503	-0.45902
H	2.44947	-0.63072	-1.52178
C	1.28892	-1.79644	-0.19473
H	0.84469	-1.74834	0.80212
H	1.90161	-2.70151	-0.19738
C	0.21600	-1.93944	-1.27608
H	-0.36421	-2.85559	-1.11312
H	0.72067	-2.07519	-2.24363
C	2.18598	1.92100	-0.89927
H	1.66386	2.87757	-0.78304
H	2.36494	1.79543	-1.97648
C	3.54090	2.00916	-0.19399
H	3.40862	2.29129	0.85835
H	4.12324	2.82365	-0.64362
C	4.35870	0.70929	-0.28430
H	4.51271	0.50366	-1.35492
C	5.75039	0.93862	0.33243
H	5.68642	1.26940	1.37485
H	6.27159	1.72673	-0.22183
H	6.38402	0.04814	0.30207
C	3.57005	-0.53563	0.26022
C	3.55017	-0.49277	1.80377
H	3.17880	0.47081	2.16925
H	4.58670	-0.58885	2.15677
O	2.76231	-1.56067	2.33570
H	2.86679	-1.54077	3.29597
C	4.33158	-1.81881	-0.15279
H	3.90003	-2.70585	0.31429
H	5.37875	-1.77279	0.15914
H	4.31236	-1.95726	-1.23951

0 Imaginary Frequencies

E = -1128.44396725

H = -1127.782447

G = -1127.863606

ΔG = 0.0

***(13S*,17R*,18R*)*-5 Conformer 2**

C	1.86126	0.77404	0.00475
H	1.30932	0.77469	0.95471
C	2.94734	1.87247	0.15406
H	2.47912	2.84133	0.35452
H	3.49451	1.98473	-0.78729
C	3.96533	1.55322	1.25253
H	3.48618	1.55323	2.23852
H	4.74141	2.32650	1.27917
C	4.63462	0.19835	0.99577
H	5.33800	-0.02530	1.80712
O	5.44749	0.26151	-0.18682
H	4.85437	0.16536	-0.94635
C	3.60144	-0.96100	0.90768
C	2.93222	-1.16129	2.29321
H	3.69317	-1.35142	3.05885
H	2.26309	-2.02635	2.26173
H	2.34375	-0.30019	2.61644
C	4.35613	-2.26377	0.58008
H	5.13608	-2.43104	1.33073
H	4.84030	-2.22501	-0.39801
H	3.68779	-3.12940	0.60262
C	2.51879	-0.60142	-0.13296
C	2.11081	-1.47470	-1.06559
H	2.61223	-2.43452	-1.15585
C	0.93771	-1.27628	-1.98766

H	0.40114	-2.22765	-2.07098
H	1.30362	-1.08067	-3.00594
C	-0.02027	-0.14156	-1.56401
H	-0.54070	0.18300	-2.47573
C	0.82630	1.10575	-1.13312
C	1.56398	1.61931	-2.39695
H	2.39341	0.97024	-2.69293
H	1.96854	2.62344	-2.24156
H	0.86762	1.67940	-3.24022
C	-1.20764	-0.59596	-0.61907
C	-0.70488	-1.30575	0.65718
H	-1.52129	-1.77960	1.20425
H	-0.19743	-0.63426	1.35159
H	0.00349	-2.09515	0.39646
C	-2.08462	0.67698	-0.34333
H	-2.33439	1.04726	-1.35083
C	-1.22692	1.78681	0.29421
H	-0.78631	1.44941	1.23946
H	-1.83858	2.65777	0.54454
C	-0.12830	2.23380	-0.67453
H	0.44892	3.05658	-0.23682
H	-0.61476	2.65180	-1.56703
C	-2.08893	-1.59858	-1.41206
H	-1.55862	-2.54808	-1.54776
H	-2.26458	-1.19519	-2.41942
C	-3.44571	-1.87423	-0.75881
H	-3.31597	-2.40710	0.19231
H	-4.01556	-2.55903	-1.39979
C	-4.27778	-0.60187	-0.53521
H	-4.41875	-0.14328	-1.52710
C	-5.67263	-0.98119	-0.01053
H	-5.61795	-1.44866	0.97650
H	-6.14177	-1.69457	-0.69788
H	-6.33850	-0.11895	0.07400
C	-3.51099	0.47446	0.30954
C	-3.41862	0.09714	1.80693
H	-2.59012	0.64938	2.27173
H	-3.20255	-0.97110	1.92498
O	-4.64287	0.41364	2.48014
H	-4.54911	0.10934	3.39235
C	-4.27511	1.81640	0.18968
H	-5.31892	1.70379	0.48273
H	-4.24611	2.17908	-0.84387
H	-3.85214	2.59233	0.83274

0 Imaginary Frequencies

E = -1128.44436172

H = -1127.782696

G = -1127.863061

ΔG = 0.3

***(13S*,17R*,18R*)*-5 Conformer 3**

C	-1.85006	0.74392	-0.25529
H	-1.33622	0.44124	-1.17822
C	-2.96986	1.71951	-0.70834
H	-2.53461	2.57770	-1.23050
H	-3.48919	2.12264	0.16679
C	-4.01392	1.04476	-1.60188
H	-3.56703	0.72901	-2.55166
H	-4.81096	1.75546	-1.84841
C	-4.63729	-0.16207	-0.89194
H	-5.36290	-0.64714	-1.55632
O	-5.40809	0.27039	0.24033
H	-4.78510	0.42966	0.96471
C	-3.56992	-1.21475	-0.47626
C	-2.94085	-1.84089	-1.74885
H	-2.25023	-2.64137	-1.46741
H	-2.38440	-1.12179	-2.35350
H	-3.72161	-2.27595	-2.38344
C	-4.27753	-2.35388	0.28255
H	-3.58587	-3.16869	0.51525
H	-5.07493	-2.76772	-0.34408
H	-4.73305	-2.00884	1.21306
C	-2.46828	-0.51772	0.35069
C	-2.01947	-1.02347	1.50865

H	-2.49633	-1.90539	1.92813
C	-0.83683	-0.50611	2.28182
H	-0.28025	-1.36353	2.67499
H	-1.19259	0.02320	3.17772
C	0.09396	0.42535	1.47502
H	0.62396	1.04538	2.21143
C	-0.78058	1.44485	0.66339
C	-1.48355	2.36341	1.69783
H	-2.29489	1.85603	2.22826
H	-1.90558	3.25343	1.22244
H	-0.76065	2.70983	2.44415
C	1.26849	-0.31176	0.71371
C	0.75766	-1.40188	-0.25022
H	0.18984	-2.15623	0.30258
H	1.58547	-1.89241	-0.76052
H	0.09524	-1.00627	-1.02112
C	2.11953	0.81019	0.02246
H	2.33567	1.50687	0.84859
C	1.24270	1.60882	-0.95893
H	0.81063	0.94981	-1.72135
H	1.83504	2.34961	-1.50502
C	0.13922	2.34917	-0.19500
H	-0.46767	2.94449	-0.88754
H	0.62517	3.07347	0.47376
C	2.18217	-0.98544	1.77226
H	1.66529	-1.83298	2.23696
H	2.38159	-0.26514	2.57938
C	3.52008	-1.46978	1.20622
H	3.36076	-2.25545	0.46227
H	4.10579	-1.92245	2.01766
C	4.34822	-0.33398	0.58285
H	4.53281	0.39127	1.39188
C	5.71838	-0.86578	0.12931
H	6.36712	-0.08006	-0.26958
H	5.60362	-1.63597	-0.63860
H	6.24237	-1.31547	0.98008
C	3.56793	0.46986	-0.51254
C	3.63482	-0.18877	-1.91098
H	4.63500	0.00184	-2.32530
H	2.91659	0.31060	-2.57607
O	3.40301	-1.59943	-1.89406
H	3.42009	-1.90442	-2.81093
C	4.29988	1.82921	-0.70024
H	4.19586	2.45261	0.19334
H	3.90951	2.39729	-1.54988
H	5.36780	1.68150	-0.88175

0 Imaginary Frequencies

E = -1128.44354073

H = -1127.781901

G = -1127.86215

ΔG = 0.9

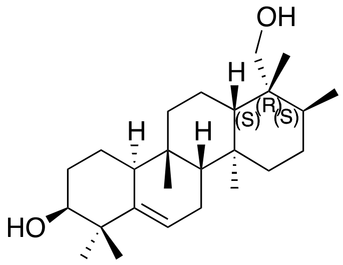

(13S,17S*,18R*)*-**5**

***(13S*,17S*,18R*)*-5 Conformer 1**

C	1.82106	0.77782	0.02563
H	1.31596	0.74132	1.00032
C	2.88271	1.90433	0.14965
H	2.39829	2.85288	0.40160
H	3.37697	2.05872	-0.81477
C	3.96563	1.58748	1.18454
H	3.53926	1.54698	2.19354
H	4.71898	2.38331	1.19208
C	4.65924	0.26109	0.85590
H	5.41291	0.03917	1.62127
O	5.40350	0.37817	-0.36706
H	4.77199	0.28688	-1.09556
C	3.65712	-0.92617	0.79391
C	3.07184	-1.18063	2.20827

H	3.87961	-1.35947	2.92730
H	2.43249	-2.06830	2.19223
H	2.47219	-0.34874	2.58287
C	4.43078	-2.19723	0.39263
H	4.85526	-2.12264	-0.61070
H	3.79197	-3.08418	0.43466
H	5.25818	-2.35482	1.09278
C	2.50831	-0.57576	-0.17612
C	2.07111	-1.44382	-1.10038
H	2.59251	-2.38720	-1.23763
C	0.84120	-1.26386	-1.94890
H	0.32239	-2.22682	-2.01009
H	1.14245	-1.05152	-2.98481
C	-0.11721	-0.15581	-1.46042
H	-0.68971	0.16757	-2.34083
C	0.72384	1.10456	-1.05383
C	1.38967	1.64569	-2.34618
H	1.77493	2.65902	-2.20243
H	0.65443	1.69296	-3.15665
H	2.22071	1.01932	-2.68346
C	-1.24558	-0.64288	-0.46124
C	-0.65798	-1.33108	0.79154
H	-1.41850	-1.87835	1.35009
H	-0.18855	-0.63364	1.48730
H	0.10208	-2.05864	0.49956
C	-2.13917	0.61482	-0.15778
H	-2.41005	0.98846	-1.15722
C	-1.28771	1.73825	0.46406
H	-0.81768	1.40788	1.39775
H	-1.91173	2.59620	0.73081
C	-0.22758	2.21106	-0.53578
H	0.35560	3.03448	-0.10774
H	-0.75235	2.63714	-1.40232
C	-2.13543	-1.68061	-1.20208
H	-1.58871	-2.62077	-1.33635
H	-2.35557	-1.30848	-2.21121
C	-3.45437	-1.98123	-0.47766
H	-3.24831	-2.46684	0.48279
H	-4.02563	-2.71564	-1.05919
C	-4.32346	-0.72719	-0.25800
H	-5.17496	-1.02295	0.37384
C	-4.94238	-0.25689	-1.58981
H	-4.19801	0.14813	-2.28208
H	-5.70299	0.51243	-1.43750
H	-5.42709	-1.10205	-2.09062
C	-3.53945	0.38468	0.52353
C	-3.41846	-0.00818	2.02109
H	-2.75959	0.70417	2.52674
H	-2.97874	-1.00184	2.14473
O	-4.65671	0.05704	2.73674
H	-5.20785	-0.67811	2.43798
C	-4.34239	1.70793	0.48286
H	-4.30071	2.17624	-0.50485
H	-3.96407	2.42947	1.21222
H	-5.39065	1.52835	0.73340

0 Imaginary Frequencies

E = -1128.44373918

H = -1127.781931

G = -1127.862627

ΔG = 0.0

***(13S*,17S*,18R*)*-5 S Conformer 2**

C	1.72250	-0.59685	0.46932
H	1.21874	-1.12763	-0.34933
C	2.76102	-1.59638	1.04296
H	2.25905	-2.50979	1.37669
H	3.23974	-1.16829	1.92952
C	3.86260	-1.95108	0.04058
H	3.44896	-2.49956	-0.81371
H	4.59935	-2.61120	0.51234
C	4.58057	-0.68783	-0.44697
H	5.34894	-0.95912	-1.18121
O	5.30568	-0.07941	0.63342
H	4.66587	0.42340	1.15846

C	3.60373	0.32333	-1.10966
C	3.04416	-0.28657	-2.42225
H	2.42758	-1.17265	-2.25765
H	3.86536	-0.57142	-3.09020
H	2.42802	0.45282	-2.94258
C	4.39854	1.58393	-1.50255
H	4.80754	2.10163	-0.63221
H	3.77908	2.28793	-2.06549
H	5.23854	1.29963	-2.14538
C	2.43139	0.61296	-0.14762
C	1.98683	1.85936	0.07177
H	2.52087	2.70202	-0.35921
C	0.73356	2.21722	0.82399
H	0.22323	3.01806	0.27733
H	1.00608	2.67520	1.78589
C	-0.22279	1.03052	1.07784
H	-0.81419	1.29274	1.96610
C	0.61867	-0.21338	1.52216
C	1.27532	0.13438	2.88384
H	2.10889	0.83554	2.78134
H	1.65531	-0.76164	3.38297
H	0.53594	0.58649	3.55368
C	-1.33063	0.81750	-0.03640
C	-0.72194	0.64727	-1.44812
H	-0.31001	-0.34912	-1.61780
H	0.08131	1.36928	-1.60774
H	-1.46199	0.82371	-2.23044
C	-2.21793	-0.39301	0.42967
H	-2.54845	-0.10039	1.43916
C	-1.35715	-1.64973	0.65393
H	-0.87129	-1.95643	-0.27544
H	-1.98780	-2.49404	0.94629
C	-0.33680	-1.40494	1.76772
H	0.24258	-2.31604	1.95768
H	-0.88885	-1.20541	2.69760
C	-2.23544	2.08278	-0.04394
H	-1.69426	2.93446	-0.47209
H	-2.46564	2.35953	0.99329
C	-3.54988	1.90363	-0.81753
H	-3.33770	1.78406	-1.88561
H	-4.13974	2.82560	-0.73902
C	-4.40024	0.71882	-0.31305
H	-5.23057	0.58512	-1.02243
C	-5.04996	1.05810	1.04278
H	-5.77919	0.30368	1.34777
H	-5.58064	2.01344	0.96683
H	-4.31911	1.15656	1.85126
C	-3.58094	-0.62222	-0.32423
C	-3.47673	-1.06803	-1.80185
H	-3.06062	-0.27563	-2.43026
H	-4.50265	-1.25217	-2.15969
O	-2.69426	-2.25692	-1.92808
H	-2.71512	-2.51623	-2.85855
C	-4.38663	-1.72931	0.39712
H	-3.98157	-2.71698	0.16898
H	-5.42959	-1.71730	0.05955
H	-4.38435	-1.60187	1.48351

0 Imaginary Frequencies

E = -1128.44237102

H = -1127.780412

G = -1127.860558

ΔG = 1.3

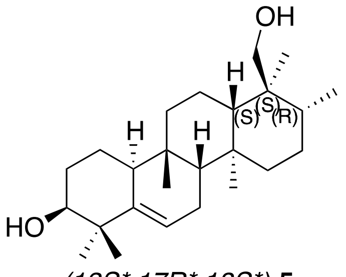

(13S,17R*,18S*)*-**5**

***(13S*,17R*,18S*)*-5 Conformer 1**

C	-1.82513	0.75876	-0.12868
H	-1.33939	0.59726	-1.10042
C	-2.87308	1.87689	-0.37332

H	-2.37898	2.78107	-0.74253
H	-3.35073	2.15314	0.57187
C	-3.97278	1.45323	-1.35024
H	-3.55909	1.28369	-2.35108
H	-4.71623	2.25256	-1.44714
C	-4.68062	0.18690	-0.85526
H	-5.44621	-0.11603	-1.57982
O	-5.40729	0.46438	0.35230
H	-4.76664	0.45296	1.07855
C	-3.69397	-0.99870	-0.65778
C	-3.12985	-1.43445	-2.03606
H	-2.50063	-2.32154	-1.91695
H	-2.52557	-0.66303	-2.51785
H	-3.94854	-1.69092	-2.71836
C	-4.47891	-2.19855	-0.09329
H	-3.85131	-3.09207	-0.03016
H	-5.31634	-2.43173	-0.75970
H	-4.89078	-1.99346	0.89714
C	-2.52853	-0.54785	0.24847
C	-2.09500	-1.29584	1.27374
H	-2.62922	-2.20453	1.53857
C	-0.85411	-1.02755	2.08206
H	-0.35217	-1.98384	2.26609
H	-1.14147	-0.67489	3.08316
C	0.11845	-0.01126	1.44306
H	0.70776	0.41494	2.26667
C	-0.70568	1.20035	0.88472
C	-1.33993	1.92376	2.10169
H	-2.17048	1.36098	2.53780
H	-1.71877	2.91198	1.82542
H	-0.58834	2.07431	2.88403
C	1.22428	-0.65040	0.50760
C	0.61526	-1.51500	-0.61749
H	-0.10490	-2.22596	-0.20558
H	1.37928	-2.09748	-1.13290
H	0.09464	-0.92648	-1.37444
C	2.12744	0.52603	-0.00459

H	2.46481	1.02712	0.91311
C	1.27874	1.57363	-0.74568
H	0.77395	1.13471	-1.61475
H	1.90965	2.37514	-1.14087
C	0.25961	2.20384	0.21003
H	-0.31304	2.98317	-0.30665
H	0.81997	2.72037	1.00186
C	2.12074	-1.56217	1.38748
H	1.56382	-2.44831	1.71266
H	2.39267	-1.01422	2.30082
C	3.40780	-2.01235	0.69076
H	3.17629	-2.67720	-0.15193
H	3.99691	-2.62092	1.38909
C	4.27388	-0.83913	0.20790
H	4.54220	-0.25138	1.09657
C	5.58224	-1.37793	-0.39490
H	6.27278	-0.58406	-0.69388
H	5.39754	-2.00871	-1.27129
H	6.10312	-1.99563	0.34499
C	3.47965	0.14447	-0.71836
C	4.31853	1.43482	-0.89757
H	3.84298	2.08249	-1.64732
H	5.30306	1.16378	-1.30156
O	4.46172	2.12418	0.34418
H	5.03684	2.88483	0.19042
C	3.30367	-0.42114	-2.14713
H	2.99591	-1.46792	-2.15373
H	4.24523	-0.36158	-2.70403
H	2.55779	0.14675	-2.71079

0 Imaginary Frequencies

E = -1128.44718865

H = -1127.785399

G = -1127.865859

ΔG = 0.0

***(13S*,17R*,18S*)*-5 Conformer 2**

C	-1.78976	0.72283	0.10952

H	-1.24619	0.80917	-0.84178
C	-2.79901	1.89996	0.11648
H	-2.26646	2.85365	0.04661
H	-3.35305	1.90854	1.05855
C	-3.81609	1.79240	-1.01957
H	-3.32283	1.85228	-1.99691
H	-4.51817	2.63654	-0.98121
C	-4.60340	0.48379	-0.91221
H	-5.29727	0.39997	-1.76433
O	-5.35229	0.45337	0.31159
H	-5.91606	1.23909	0.32208
C	-3.68424	-0.76471	-0.93033
C	-3.07489	-0.90680	-2.35149
H	-2.49501	-1.83225	-2.41373
H	-2.40894	-0.08200	-2.61599
H	-3.86715	-0.95242	-3.10780
C	-4.55663	-2.01112	-0.68364
H	-4.96988	-2.02100	0.32633
H	-3.98909	-2.93309	-0.84025
H	-5.39327	-2.01962	-1.39117
C	-2.53279	-0.61564	0.08585
C	-2.12506	-1.64256	0.84372
H	-2.67930	-2.57718	0.82276
C	-0.90338	-1.65020	1.72312
H	-0.40344	-2.61866	1.61005
H	-1.21657	-1.62755	2.77734
C	0.08342	-0.49030	1.46192
H	0.63705	-0.33454	2.39874
C	-0.72889	0.83519	1.26287
C	-1.44135	1.14908	2.60383
H	-0.74155	1.04195	3.43988
H	-2.29272	0.48729	2.78578
H	-1.81172	2.17815	2.62384
C	1.23050	-0.82221	0.42106
C	0.67366	-1.30367	-0.93727
H	-0.10696	-2.05253	-0.78629
H	1.44951	-1.76760	-1.54770
H	0.24018	-0.49742	-1.53170
C	2.14677	0.44832	0.32588
H	2.45114	0.62750	1.37064
C	1.32249	1.68376	-0.07707
H	0.85310	1.53181	-1.05688
H	1.97720	2.54952	-0.19297
C	0.26009	1.99054	0.98295
H	-0.29043	2.89981	0.71450
H	0.77859	2.22554	1.92389
C	2.10027	-1.95923	1.02048
H	1.54000	-2.90091	1.04576
H	2.33608	-1.71121	2.06521
C	3.41008	-2.18413	0.26272
H	3.20637	-2.56686	-0.74551
H	3.98302	-2.97465	0.76405
C	4.29376	-0.92982	0.16181
H	4.56689	-0.65126	1.19277
C	5.59331	-1.30351	-0.57491
H	6.29043	-0.46793	-0.68026
H	5.38471	-1.68990	-1.57812
H	6.11522	-2.09377	-0.02431
C	3.52744	0.30911	-0.42790
C	4.39994	1.54917	-0.08042
H	5.45827	1.29503	-0.21663
H	4.26135	1.79907	0.98282
O	4.08647	2.67508	-0.90704
H	4.68538	3.38965	-0.65306
C	3.40549	0.22040	-1.96386
H	4.38180	0.36610	-2.43574
H	2.74409	0.99667	-2.35316
H	3.02193	-0.74638	-2.29382

0 Imaginary Frequencies

E = -1128.44341691

H = -1127.782273

G = -1127.863607

ΔG = 1.4

***(13S*,17R*,18S*)*-5 Conformer 3**

C	-1.86095	0.75887	0.02820
H	-1.32771	0.76507	-0.93292
C	-2.88581	1.91983	-0.06109
H	-2.36748	2.87113	-0.21829
H	-3.43472	2.00386	0.88024
C	-3.90803	1.69957	-1.17643
H	-3.42139	1.68038	-2.15865
H	-4.62132	2.53455	-1.20661
C	-4.67689	0.39535	-0.94896
H	-5.37343	0.22677	-1.78622
O	-5.42030	0.46529	0.27702
H	-5.98876	1.24577	0.22273
C	-3.74160	-0.83855	-0.86010
C	-3.13851	-1.10079	-2.26689
H	-2.54297	-2.01810	-2.24890
H	-2.48870	-0.29287	-2.61104
H	-3.93463	-1.22840	-3.00956
C	-4.59692	-2.06815	-0.49717
H	-5.00478	-1.99237	0.51209
H	-4.01911	-2.99383	-0.57453
H	-5.43712	-2.14907	-1.19577
C	-2.58761	-0.58426	0.13134
C	-2.16605	-1.53181	0.97947
H	-2.70899	-2.47076	1.04695
C	-0.94391	-1.44211	1.85352
H	-0.43727	-2.41313	1.83927
H	-1.25730	-1.31522	2.90017
C	0.03386	-0.30702	1.47770
H	0.59030	-0.05957	2.39278
C	-0.78947	0.98781	1.15495
C	-1.48767	1.43264	2.46548
H	-2.33394	0.79005	2.72453
H	-1.86142	2.45754	2.38735
H	-0.77716	1.41125	3.29908
C	1.18054	-0.72595	0.46768
C	0.62073	-1.31170	-0.84849
H	-0.13517	-2.06945	-0.63088
H	1.40127	-1.79403	-1.43753
H	0.15467	-0.56295	-1.49046
C	2.09900	0.53573	0.26806
H	2.37640	0.82509	1.29594
C	1.24880	1.70261	-0.27632
H	0.77592	1.42646	-1.22515
H	1.86171	2.57956	-0.50042
C	0.18830	2.11542	0.74909
H	-0.37178	2.98326	0.38256
H	0.70839	2.45785	1.65489
C	2.04095	-1.82184	1.14834
H	1.46805	-2.75035	1.24961
H	2.28953	-1.49721	2.16902
C	3.33936	-2.11676	0.39890
H	3.12666	-2.55937	-0.58278
H	3.90399	-2.88225	0.94675
C	4.23742	-0.88238	0.22849
H	4.48798	-0.54070	1.24567
C	5.55268	-1.31207	-0.44544
H	6.25432	-0.48384	-0.53591
H	5.37116	-1.71510	-1.44831
H	6.02207	-2.10947	0.14261
C	3.49669	0.32907	-0.44737
C	4.30669	1.62424	-0.15505
H	4.45915	1.70480	0.93294
H	3.73313	2.50285	-0.46975
O	5.56505	1.63286	-0.83546
H	5.94384	2.51406	-0.72116
C	3.38423	0.16489	-1.97734
H	2.72637	0.92647	-2.40796
H	2.99185	-0.81189	-2.26677
H	4.36543	0.28214	-2.44155

0 Imaginary Frequencies

E = -1128.44247097

H = -1127.781332

G = -1127.862949

ΔG = 1.8

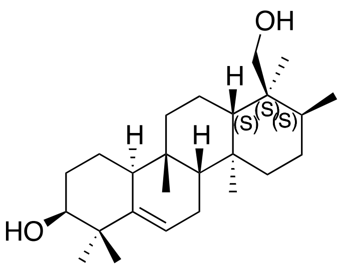

(13S,17S*,18S*)*-**5**

***(13S*,17S*,18S*)*-5 Conformer 1**

C	1.82551	-0.76971	0.11738
H	1.34838	-0.89233	-0.86521
C	2.84332	-1.93267	0.23182
H	2.32494	-2.89502	0.16948
H	3.34011	-1.89801	1.20426
C	3.92354	-1.86149	-0.84907
H	3.48279	-1.96120	-1.84724
H	4.63282	-2.68871	-0.73202
C	4.69433	-0.54688	-0.75850
H	5.41690	-0.48800	-1.58831
O	5.40597	-0.59096	0.48581
H	6.00687	0.16461	0.50983
C	3.76152	0.69828	-0.86038
C	3.23220	0.79937	-2.31629
H	2.64294	1.71317	-2.43658
H	2.59631	-0.04335	-2.59720
H	4.06575	0.83886	-3.02736
C	4.60906	1.95944	-0.60008
H	4.93555	2.02848	0.44179
H	4.05288	2.87212	-0.83093
H	5.49702	1.95356	-1.24340
C	2.56215	0.57089	0.10022
C	2.11966	1.61040	0.82054
H	2.67108	2.54686	0.80750
C	0.85853	1.63026	1.64207
H	0.36940	2.60034	1.50083
H	1.12161	1.61157	2.70987
C	-0.12229	0.47526	1.34120
H	-0.72597	0.33540	2.24906
C	0.68876	-0.85917	1.20037
C	1.30622	-1.17204	2.58825
H	1.67298	-2.20135	2.63641
H	0.55003	-1.06117	3.37299
H	2.14386	-0.51138	2.82895
C	-1.20918	0.80157	0.23735
C	-0.57190	1.24311	-1.09978
H	-1.30486	1.70328	-1.76280
H	-0.11448	0.41987	-1.65120
H	0.20883	1.98490	-0.91890
C	-2.13260	-0.46552	0.12381
H	-2.46271	-0.64697	1.15866
C	-1.29691	-1.70486	-0.24671
H	-0.78943	-1.56401	-1.20821
H	-1.93435	-2.58469	-0.37488
C	-0.28493	-2.01382	0.86292
H	0.28039	-2.92003	0.61668
H	-0.85194	-2.25542	1.77324
C	-2.08711	1.97321	0.75975
H	-1.51342	2.90698	0.75361
H	-2.34752	1.78273	1.80935
C	-3.37393	2.18368	-0.05062
H	-3.12147	2.50721	-1.06644
H	-3.94395	3.01210	0.38872
C	-4.27056	0.93216	-0.10776
H	-5.07850	1.13624	-0.82091
C	-4.95167	0.68585	1.25330
H	-5.44196	1.60573	1.59160
H	-4.24831	0.38907	2.03760
H	-5.71860	-0.08759	1.17391
C	-3.47766	-0.29888	-0.67435
C	-4.31372	-1.58688	-0.46938
H	-4.34119	-1.85635	0.59646
H	-3.84604	-2.42100	-1.00754
O	-5.63867	-1.38244	-0.96924

H -6.09040 -2.23579 -0.94429

C -3.30119 -0.14522 -2.20384

H -4.26194 -0.33269 -2.69197

H -2.57999 -0.86837 -2.59840

H -2.96973 0.84958 -2.50281

0 Imaginary Frequencies

E = -1128.44284951

H = -1127.781365

G = -1127.861745

ΔG = 0.0

***(13S*,17S*,18S*)*-5Conformer 2**

C 1.76003 0.75138 0.01776

H 1.31336 0.67000 1.01851

C 2.75130 1.94091 0.10486

H 2.21660 2.85770 0.37340

H 3.21515 2.11366 -0.86926

C 3.86793 1.68493 1.11830

H 3.45737 1.57056 2.12771

H 4.55496 2.53816 1.14988

C 4.66164 0.43696 0.74295

H 5.41687 0.23563 1.51977

O 5.32493 0.74736 -0.49043

H 5.94392 0.03143 -0.68190

C 3.76103 -0.82964 0.62625

C 3.29461 -1.23164 2.05164

H 2.72914 -2.16706 2.00991

H 2.65389 -0.47865 2.51670

H 4.15791 -1.38687 2.70954

C 4.62553 -1.98828 0.08965

H 4.90543 -1.84193 -0.95779

H 4.10361 -2.94637 0.15970

H 5.54118 -2.07763 0.68622

C 2.51789 -0.55201 -0.24359

C 2.05326 -1.45735 -1.11558

H 2.61712 -2.36934 -1.29367

C 0.75257 -1.36041 -1.86682

H 0.27876 -2.34849 -1.86465

H 0.96218 -1.16197 -2.92823

C -0.22053 -0.28723 -1.33194

H -0.86357 -0.00273 -2.17652

C 0.58728 1.01522 -0.99532

C 1.16091 1.56098 -2.32869

H 1.51470 2.58970 -2.21572

H 0.38390 1.56928 -3.10085

H 1.99852 0.96107 -2.69549

C -1.25865 -0.80659 -0.25500

C -0.56385 -1.44917 0.96728

H -1.26466 -2.01659 1.58097

H -0.08489 -0.72005 1.62279

H 0.20896 -2.14841 0.63978

C -2.18495 0.41371 0.08976

H -2.55373 0.75602 -0.88591

C -1.35511 1.59000 0.63308

H -0.81618 1.30863 1.54562

H -2.00910 2.42210 0.90900

C -0.38223 2.08734 -0.44073

H 0.18546 2.94838 -0.06968

H -0.98122 2.46603 -1.28063

C -2.15099 -1.89206 -0.92229

H -1.57292 -2.80817 -1.08913

H -2.45714 -1.54001 -1.91605

C -3.40021 -2.24720 -0.10354

H -3.10177 -2.72440 0.83644

H -3.98073 -3.00549 -0.64434

C -4.30646 -1.03286 0.18631

H -5.06867 -1.35711 0.91061

C -5.07088 -0.62451 -1.08855

H -5.63349 -1.48725 -1.46406

H -4.40500 -0.29161 -1.88978

H -5.77656 0.18664 -0.90250

C -3.49644 0.11310 0.89024

C -4.35774 1.39965 0.99150

H -3.95864 2.03649 1.79404

H -5.37374 1.10342 1.29689

O -4.39156 2.13068 -0.23372

H -4.93889 2.91312 -0.08613

C -3.26609 -0.28320 2.37032

H -4.20748 -0.20027 2.92752

H -2.53895 0.37471 2.85523

H -2.91759 -1.30893 2.48830

0 Imaginary Frequencies

E = -1128.44210509

H = -1127.780214

G = -1127.860013

ΔG = 1.1

***(13S*,17S*,18S*)*-5Conformer 3**

C -1.75032 0.71935 0.21555

H -1.26979 0.93601 -0.74867

C -2.74301 1.88582 0.45454

H -2.20381 2.83792 0.49231

H -3.24128 1.76035 1.41904

C -3.82269 1.95296 -0.62741

H -3.37686 2.14585 -1.60953

H -4.51268 2.77988 -0.42475

C -4.62326 0.65459 -0.67433

H -5.34500 0.69864 -1.50579

O -5.33720 0.58636 0.56773

H -5.95761 -0.15158 0.51284

C -3.71966 -0.59516 -0.90585

C -3.19575 -0.55696 -2.36680

H -2.63372 -1.46942 -2.58617

H -2.53500 0.29191 -2.55779

H -4.03102 -0.49550 -3.07437

C -4.59839 -1.85499 -0.77220

H -4.91928 -2.02415 0.25994

H -4.06973 -2.75261 -1.10407

H -5.49017 -1.75676 -1.40254

C -2.51326 -0.59706 0.05357

C -2.08210 -1.72157 0.64142

H -2.65108 -2.64053 0.52604

C -0.81125 -1.85938 1.43664

H -0.33740 -2.81012 1.16811

H -1.06160 -1.97207 2.50173

C 0.18082 -0.68719 1.27004

H 0.79271 -0.66626 2.18276

C -0.61530 0.66345 1.30100

C -1.23529 0.80129 2.71595

H -1.57629 1.82371 2.90243

H -0.48787 0.56559 3.48151

H -2.09053 0.13519 2.86045

C 1.25746 -0.88465 0.12429

C 0.60593 -1.13558 -1.25562

H 1.32029 -1.54176 -1.97284

H 0.18323 -0.23376 -1.70203

H -0.20264 -1.86399 -1.16485

C 2.20022 0.37432 0.17677

H 2.53291 0.41013 1.22632

C 1.38399 1.66401 -0.02391

H 0.87362 1.65427 -0.99467

H 2.05045 2.52825 -0.05010

C 0.37567 1.83763 1.11584

H -0.17605 2.77686 0.99269

H 0.94239 1.94826 2.05158

C 2.11354 -2.13228 0.48012

H 1.52653 -3.04715 0.34040

H 2.37233 -2.09710 1.54652

C 3.40130 -2.24896 -0.34756

H 3.15256 -2.42585 -1.39945

H 3.95794 -3.13785 -0.02458

C 4.31430 -1.01425 -0.22554

H 5.12986 -1.13349 -0.95367

C 4.97905 -0.98055 1.16773

H 5.45116 -1.94812 1.37029

H 4.26520 -0.79329 1.97555

H 5.76207 -0.22095 1.23686

C	3.54902	0.29786	-0.63043
C	4.46843	1.49000	-0.25619
H	5.50336	1.21717	-0.51635
H	4.43695	1.66642	0.82801
O	4.10635	2.68112	-0.96159
H	4.69670	3.38208	-0.65519
C	3.38154	0.33324	-2.16629
H	4.35983	0.46427	-2.64261
H	2.75318	1.16847	-2.48109
H	2.94713	-0.58440	-2.56131

0 Imaginary Frequencies

E = -1128.44131415

H = -1127.77944

G = -1127.859136

ΔG = 1.6

Theoretical Prediction of Optical Rotation of 5

The optical rotation of **5** was predicted using the method of Stephens et al.[15] According to this method, the absolute configuration of a conformationally rigid molecule may be predicted with 95% confidence if the specific rotation calculated at the TD-B3LYP/aug-cc-pVDZ//B3LYP/6-31G(d) level of theory for one, but not both, of the enantiomers lies within ±57.8 of the experimental value. (The value of ±57.8 corresponds to $\pm 2\sigma$ from a benchmark set of $[\alpha]_D$ values for 65 molecules.). For **5**, the experimental $[\alpha]_D$ is +32 (*c* 0.23, $CHCl_3$), and therefore, for its absolute stereochemistry to be predicted with 95% confidence, the computed $[\alpha]_D$ for one enantiomer must lie within the range –25.8 to +89.8 and the value for the other enantiomer must lie outside this range.

The computed $[\alpha]_D$ values for the enantiomers of **5** are shown in Figure S2. For the absolute stereochemistry corresponding to that drawn in Figure 1 of the paper (**5**), the computed $[\alpha]_D$ value is +73. The $[\alpha]_D$ value for the opposite enantiomer (*ent*-**5**) is –73. These values permit the absolute stereochemistry of **5** to be assigned with 95% confidence as that shown in Figures 1 and S2.

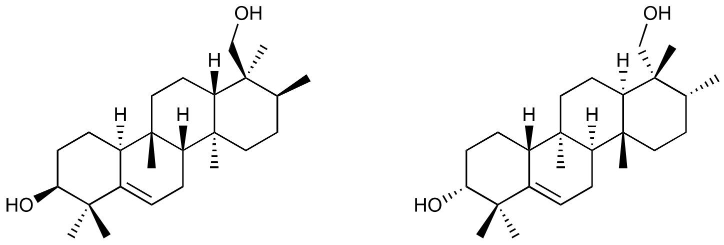

5

Expt: $[\alpha]_D$ +32 (*c* 0.23, $CHCl_3$)
Calc: $[\alpha]_D$ +73

ent-**5**

Calc: $[\alpha]_D$ -73

Figure S2. Comparison of experimental and calculated $[\alpha]_D$ values for the enantiomers of **5**.

*Optimized geometry of **5** at the B3LYP/6-31G(d) level of theory*

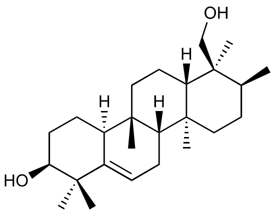

5

C	1.827669	-0.769794	0.112803
H	1.350300	-0.892606	-0.870720
C	2.847121	-1.931786	0.228239
H	2.329010	-2.895547	0.166014
H	3.347432	-1.894980	1.198908
C	3.926275	-1.860678	-0.854113
H	3.486371	-1.959685	-1.854108
H	4.637346	-2.686532	-0.734328
C	4.697142	-0.546360	-0.757597
H	5.422928	-0.484829	-1.586839
O	5.401083	-0.603300	0.486041
H	5.955477	0.187897	0.551732
C	3.765425	0.700518	-0.857362
C	3.241817	0.811420	-2.314494
H	2.649503	1.724821	-2.432109
H	2.607811	-0.031457	-2.602953
H	4.077795	0.856504	-3.024388
C	4.613798	1.960130	-0.588557
H	4.924831	2.032680	0.459333
H	4.063866	2.875537	-0.828214
H	5.511120	1.952131	-1.221542
C	2.563341	0.571590	0.098678
C	2.119535	1.609682	0.819606
H	2.670848	2.547016	0.811123
C	0.858876	1.628126	1.642153
H	0.368662	2.599154	1.504762
H	1.123741	1.604719	2.710228
C	-0.122028	0.473566	1.338576
H	-0.725589	0.332463	2.247260
C	0.690887	-0.859978	1.195917
C	1.310699	-1.173922	2.582737
H	1.671778	-2.206210	2.631485
H	0.557344	-1.057830	3.371115
H	2.154682	-0.518448	2.819185
C	-1.209679	0.801368	0.235155
C	-0.573308	1.245617	-1.101637
H	-1.307074	1.706851	-1.764321
H	-0.115185	0.423590	-1.656237
H	0.207871	1.988303	-0.920314
C	-2.133138	-0.466307	0.121859
H	-2.461485	-0.648705	1.158026
C	-1.296624	-1.705231	-0.250014
H	-0.790323	-1.563835	-1.212872
H	-1.933842	-2.586701	-0.377695
C	-0.282741	-2.014649	0.858397
H	0.282449	-2.921375	0.609917
H	-0.848907	-2.258613	1.769782
C	-2.088611	1.972341	0.758537
H	-1.516183	2.907968	0.749823
H	-2.346731	1.783557	1.809947
C	-3.378373	2.181214	-0.048551
H	-3.129211	2.507823	-1.065068
H	-3.948070	3.008946	0.394532

C	-4.274500	0.929122	-0.104996
H	-5.085109	1.131290	-0.815845
C	-4.953689	0.681788	1.257283
H	-5.437749	1.604535	1.599858
H	-4.251293	0.377529	2.041366
H	-5.728664	-0.084330	1.174747
C	-3.479699	-0.299922	-0.674079
C	-4.315511	-1.587988	-0.469075
H	-4.338897	-1.861796	0.597021
H	-3.852225	-2.421241	-1.015108
O	-5.640133	-1.374281	-0.961724
H	-6.101407	-2.225643	-0.940745
C	-3.309708	-0.146425	-2.204518
H	-4.273958	-0.329914	-2.688982
H	-2.590752	-0.871336	-2.603503
H	-2.975968	0.848301	-2.504483

0 imaginary frequencies
E = -1128.367853

Carbocation Rearrangements Leading to 5 and 6

Density functional theory calculations at the B3LYP/6-31+G(d,p) level of theory in the gas phase were performed to investigate the proposed biosynthetic pathways leading to **5** and **6** (Figure 2). The global minimum conformations of the C-18 epimeric carbocations α-**C** and β-**C** were identified as D-ring chair conformers by means of conformational searching. Transition states for 1,2 hydride migrations from C-18 to C-17 in α-**C** and β-**C** were computed. Intrinsic Reaction Coordinate (IRC)[16] calculations were performed to determine the conformation of the reactant immediately preceding each TS. For α-**C**, the IRC path back to reactants led directly to the global minimum conformer, while for β-**C**, the IRC led to a twist-boat conformer that lay 4.7 kcal/mol above the global minimum. The boat transition state for cyclization of **B** to form β-**C** was also calculated. An IRC calculation on this TS indicated that the D ring in the immediate product adopted the twist-boat conformation.

Optimized geometries at the B3LYP/6-31+G(d,p) level of theory

Gibbs free energies (G) for these species are reported at a standard state of 1 atm (25 °C).

α-C

C	-2.492677	-1.662981	0.851720
C	-1.962166	-0.822866	-0.337223
C	-2.648475	0.581517	-0.235891
C	-4.214617	0.617104	-0.273221
C	-4.685940	-0.324711	0.873133
C	-4.032772	-1.713386	0.868305
C	-1.902121	1.581136	-1.130206
C	-0.505678	1.906385	-0.541189
C	0.179643	0.720261	0.232059
C	-0.383832	-0.620082	-0.347201
C	1.724051	0.746737	-0.138485
C	2.560644	-0.498829	0.293350
C	1.882418	-1.784280	-0.228017
C	0.394186	-1.841069	0.167081
C	2.429675	2.051110	0.244001
C	3.872886	2.127516	-0.413390
C	4.567277	0.890979	-0.034662
C	4.001237	-0.356081	-0.536131
C	4.917848	-1.580818	-0.487348
O	6.116149	-1.194955	-1.159762
C	2.847424	-0.627389	1.802666
C	5.694338	0.946855	0.904341
C	-0.037878	0.896207	1.755805
C	-2.256932	-1.582592	-1.657998

C	-4.691382	2.045883	0.088732
C	-4.864366	0.253525	-1.626253
O	-6.109762	-0.428929	0.796417
H	4.394194	3.024528	-0.073091
H	3.728321	2.170337	-1.498271
H	-3.252990	-2.019035	-1.689549
H	-1.550046	-2.410886	-1.781332
H	-2.155206	-0.934204	-2.533569
H	-2.107976	-2.688806	0.813675
H	-2.139811	-1.231186	1.797976
H	-4.362251	-2.259410	1.762390
H	-4.414147	-2.280542	0.012929
H	-4.542896	2.746980	-0.738190
H	-4.165632	2.439934	0.967571
H	-5.760411	2.027137	0.314939
H	-4.826561	-0.810260	-1.859632
H	-4.381291	0.798706	-2.444558
H	-5.919364	0.536295	-1.613300
H	-2.451062	0.909900	0.790696
H	-2.455472	2.517471	-1.234644
H	-1.797614	1.180165	-2.145196
H	-0.596600	2.761223	0.139863
H	0.138154	2.240269	-1.364492
H	0.220944	0.009046	2.335182
H	0.550977	1.731757	2.147655
H	-1.077814	1.126127	1.983557
H	-0.137933	-0.549278	-1.417975
H	1.716929	0.711295	-1.237407
H	1.964668	-0.982079	2.332330
H	3.151027	0.310829	2.274957
H	3.633693	-1.363614	1.990951
H	2.399413	-2.670802	0.155689
H	1.950537	-1.817115	-1.323166
H	0.287380	-1.951886	1.251320
H	-0.015663	-2.759937	-0.264408
H	2.527839	2.171253	1.325901
H	1.893420	2.927622	-0.124973
H	5.668845	1.833316	1.543086
H	6.582902	1.039334	0.251054
H	5.846278	0.031276	1.477296
H	4.426278	-2.411518	-1.003045
H	5.132488	-1.899890	0.540549
H	6.664284	-1.971308	-1.336451
H	3.674607	-0.208102	-1.569900
H	-4.411959	0.174640	1.819215
H	-6.438311	-0.909293	1.567263

0 imaginary frequencies
E = -1128.802623
G = -1128.212888

TS: 1,2-Hydride Shift from C-18 to C-17 in α-C

C	-2.503919	-1.691718	0.750563
C	-1.911057	-0.792634	-0.363447
C	-2.602820	0.605453	-0.224736
C	-4.164334	0.646638	-0.346859
C	-4.698306	-0.353716	0.719734
C	-4.042681	-1.740905	0.681179
C	-1.807426	1.649941	-1.020325
C	-0.448098	1.940208	-0.334490
C	0.196624	0.711165	0.406361
C	-0.337393	-0.590091	-0.276586
C	1.748486	0.766795	0.132018

C	2.587752	-0.498861	0.527799
C	1.919757	-1.763266	-0.087211
C	0.415392	-1.835900	0.213711
C	2.445491	2.044604	0.627219
C	3.804136	2.217544	-0.059769
C	4.569171	0.917960	-0.303560
C	4.017084	-0.340181	0.065069
C	4.909283	-1.575543	-0.084441
O	5.147533	-1.716111	-1.481831
C	2.806765	-0.718872	2.068502
C	6.055487	1.101871	-0.577717
C	-0.102603	0.803577	1.924766
C	-2.132726	-1.482837	-1.735795
C	-4.661771	2.055175	0.062913
C	-4.736918	0.356286	-1.751489
O	-6.115358	-0.454003	0.556133
H	4.469071	2.853757	0.536027
H	3.696323	2.731574	-1.022777
H	-3.124770	-1.915785	-1.844945
H	-1.419140	-2.305090	-1.862558
H	-1.983220	-0.790941	-2.570467
H	-2.116321	-2.714958	0.681869
H	-2.203701	-1.307993	1.734901
H	-4.376089	-2.264766	-0.220655
H	-4.419533	-2.330330	1.527800
H	-4.468080	2.798049	-0.716777
H	-4.185034	2.400980	0.988714
H	-5.741682	2.027042	0.228616
H	-4.679931	-0.692735	-2.040926
H	-4.212537	0.947136	-2.510597
H	-5.792664	0.635218	-1.781516
H	-2.461270	0.876703	0.827687
H	-2.354842	2.591529	-1.107400
H	-1.644328	1.302328	-2.047099
H	-0.581151	2.755356	0.386574
H	0.241650	2.321332	-1.098117
H	0.078169	-0.131172	2.459367
H	0.503082	1.581434	2.400696
H	-1.141789	1.072198	2.110829
H	-0.032319	-0.462026	-1.326934
H	1.811000	0.787783	-0.969567
H	1.870164	-1.044219	2.518505
H	3.139276	0.184961	2.581285
H	3.539801	-1.508772	2.262834
H	2.399581	-2.674015	0.285221
H	2.062813	-1.748694	-1.175253
H	0.248044	-2.002718	1.283526
H	0.030830	-2.730596	-0.285821
H	2.581037	2.018465	1.712771
H	1.832532	2.923984	0.420772
H	6.204046	1.974452	-1.218539
H	6.517447	0.240054	-1.056585
H	6.560459	1.304988	0.373503
H	4.409012	-2.452497	0.328518
H	5.841737	-1.408722	0.472494
H	5.575850	-2.565200	-1.661597
H	4.084429	0.359427	-1.268380
H	-4.481183	0.095725	1.704652
H	-6.486432	-0.968488	1.284469

1 imaginary frequency

E = -1128.782887

G = -1128.196004

α-D

C	-2.513780	-1.687423	0.764734
C	-1.923911	-0.796388	-0.357450
C	-2.613554	0.603262	-0.226025
C	-4.175447	0.645071	-0.342809
C	-4.706972	-0.347794	0.732064
C	-4.052746	-1.735874	0.700429
C	-1.819297	1.641061	-1.031418
C	-0.460084	1.936802	-0.348627
C	0.191477	0.709857	0.390220
C	-0.349080	-0.595355	-0.278097
C	1.742542	0.769943	0.098814
C	2.587652	-0.498156	0.461990
C	1.899519	-1.783017	-0.099669
C	0.401251	-1.835431	0.227645
C	2.439607	2.037584	0.619345
C	3.823695	2.219010	-0.019374
C	4.548675	0.910718	-0.423236
C	3.989566	-0.359135	0.018497
C	4.887664	-1.584187	-0.077764
O	5.254122	-1.683930	-1.449173
C	2.871339	-0.702650	2.019331
C	6.077015	1.080596	-0.466396
C	-0.100140	0.807018	1.910356
C	-2.150478	-1.496289	-1.723944
C	-4.669837	2.056747	0.059765
C	-4.753236	0.346406	-1.743553
O	-6.124392	-0.448005	0.573318
H	4.491638	2.749703	0.667685
H	3.756949	2.846683	-0.913699
H	-3.143948	-1.927107	-1.827710
H	-1.439523	-2.321714	-1.845924
H	-2.000672	-0.811185	-2.564090
H	-2.127567	-2.711628	0.701840
H	-2.210408	-1.297408	1.745644
H	-4.389283	-2.265182	-0.197014
H	-4.427339	-2.319607	1.551921
H	-4.477880	2.794596	-0.725072
H	-4.189851	2.407791	0.981910
H	-5.749227	2.030842	0.229079
H	-4.698230	-0.704446	-2.026739
H	-4.231264	0.932184	-2.508200
H	-5.808857	0.625936	-1.771417
H	-2.468112	0.881785	0.824107
H	-2.366443	2.582131	-1.125252
H	-1.657245	1.285458	-2.055637
H	-0.595515	2.750425	0.373549
H	0.226821	2.322150	-1.112358
H	0.080647	-0.126360	2.449011
H	0.504213	1.587487	2.382563
H	-1.139573	1.073096	2.098693
H	-0.046300	-0.481315	-1.330596
H	1.797346	0.809490	-1.001242
H	1.904193	-0.876659	2.489941
H	3.332126	0.165143	2.492460
H	3.491913	-1.582074	2.207475
H	2.383214	-2.683670	0.288660
H	2.024953	-1.793546	-1.189299
H	0.249277	-1.980715	1.302898
H	0.006680	-2.738514	-0.249109
H	2.536341	1.998541	1.708284
H	1.831600	2.918828	0.406145
H	6.313964	2.010526	-0.988835

```
H    6.579326    0.264555   -0.984196
H    6.469968    1.168537    0.552091
H    4.376209   -2.483155    0.268165
H    5.761671   -1.406447    0.569407
H    5.738304   -2.509146   -1.595184
H    4.224271    0.661474   -1.483430
H   -4.486324    0.107847    1.713357
H   -6.494545   -0.954098    1.307979
0 imaginary frequencies
E = -1128.783959
G = -1128.196906
```

β-C (global minimum: D ring chair conformation)

```
C    2.551250   -1.633267   -0.846960
C    1.934883   -0.815936    0.316032
C    2.552267    0.621158    0.230657
C    4.111425    0.737098    0.327034
C    4.674003   -0.184870   -0.794232
C    4.091428   -1.605118   -0.806621
C    1.721283    1.585952    1.087983
C    0.335633    1.835712    0.440270
C   -0.257966    0.610195   -0.347676
C    0.348575   -0.695972    0.265503
C   -1.817288    0.566409   -0.040200
C   -2.571131   -0.726581   -0.480737
C   -1.849228   -1.966524    0.088281
C   -0.348964   -1.961712   -0.261193
C   -2.570112    1.826684   -0.477194
C   -4.035939    1.846425    0.142265
C   -4.663823    0.602450   -0.299466
C   -4.080438   -0.673270    0.145301
C   -4.188549   -0.811654    1.680017
O   -5.537500   -0.451697    1.980672
C   -2.758994   -0.899002   -2.005500
C   -5.783006    0.622258   -1.244191
C    0.014189    0.784177   -1.863574
C    2.218795   -1.552559    1.652587
C    4.527009    2.186948   -0.026764
C    4.727554    0.414229    1.705696
O    6.097273   -0.217060   -0.661196
H   -4.581656    2.733398   -0.184142
H   -3.916400    1.864206    1.229631
H    3.233368   -1.938928    1.724223
H    1.549632   -2.414027    1.756697
H    2.053417   -0.904384    2.518749
H    2.217883   -2.676850   -0.816304
H    2.212065   -1.223954   -1.807920
H    4.481542   -2.136407   -1.684943
H    4.469107   -2.149082    0.065279
H    4.310479    2.883847    0.788741
H    4.015137    2.547878   -0.927718
H    5.603477    2.223501   -0.212016
H    4.740529   -0.649431    1.941874
H    4.183854    0.933429    2.502582
H    5.764985    0.755144    1.731669
H    2.377893    0.932754   -0.805222
H    2.221871    2.549461    1.209367
H    1.596150    1.184109    2.100437
H    0.412103    2.688488   -0.245212
H   -0.357035    2.144319    1.233455
H   -0.122494   -0.134640   -2.435304
H   -0.635354    1.548100   -2.302557
```

H	1.035526	1.115749	-2.046867
H	0.060176	-0.628367	1.325910
H	-1.846913	0.559447	1.057464
H	-3.478137	-1.700172	-2.207057
H	-1.829469	-1.185068	-2.493099
H	-3.120132	0.003922	-2.507419
H	-2.329231	-2.880486	-0.282877
H	-1.930132	-1.982726	1.180946
H	-0.200864	-2.077797	-1.339593
H	0.094373	-2.852714	0.194474
H	-2.641124	1.920627	-1.563668
H	-2.091244	2.736681	-0.109325
H	-5.880898	1.556287	-1.801615
H	-6.665182	0.543909	-0.575318
H	-5.823853	-0.257518	-1.891199
H	-3.480997	-0.157938	2.203005
H	-3.982786	-1.848411	1.962073
H	-5.749548	-0.667219	2.899303
H	-4.638475	-1.504637	-0.294961
H	4.413400	0.296064	-1.753405
H	6.479629	-0.679883	-1.417845

0 imaginary frequencies
E = -1128.801141
G = -1128.211933

β-C (reactive conformer: D ring twist-boat conformation)

C	-2.435646	-1.770321	0.567112
C	-1.827650	-0.757874	-0.435811
C	-2.539394	0.612925	-0.178574
C	-4.099713	0.650400	-0.322544
C	-4.640449	-0.457024	0.628456
C	-3.971348	-1.827028	0.457263
C	-1.751860	1.739422	-0.865332
C	-0.385642	1.974498	-0.165944
C	0.226591	0.704497	0.525805
C	-0.260166	-0.546581	-0.283888
C	1.783409	0.761174	0.319532
C	2.594458	-0.508941	0.715613
C	2.026103	-1.692070	-0.102022
C	0.502386	-1.824329	0.103934
C	2.493841	2.025919	0.841830
C	3.744702	2.228936	-0.014944
C	4.457356	0.992854	-0.396373
C	4.167883	-0.234748	0.347974
C	4.996094	-1.468440	-0.035874
O	4.791726	-1.740395	-1.417338
C	2.601502	-0.875803	2.217935
C	5.480482	1.076004	-1.448641
C	-0.141338	0.701275	2.033781
C	-2.005611	-1.312311	-1.874826
C	-4.619507	2.005175	0.220377
C	-4.644960	0.498155	-1.758723
O	-6.054282	-0.549741	0.434299
H	4.511698	2.861533	0.473385
H	3.525941	2.781832	-0.942038
H	-2.990067	-1.740905	-2.053134
H	-1.278702	-2.110849	-2.061958
H	-1.842464	-0.539851	-2.632596
H	-2.032210	-2.776446	0.403622
H	-2.162863	-1.485636	1.592212
H	-4.279546	-2.253810	-0.502713

H	-4.360177	-2.506284	1.227803
H	-4.421551	2.826741	-0.474789
H	-4.161865	2.257821	1.185132
H	-5.701647	1.950318	0.363647
H	-4.575816	-0.517328	-2.148103
H	-4.110644	1.163322	-2.445904
H	-5.701895	0.773254	-1.780875
H	-2.417243	0.782857	0.896389
H	-2.309577	2.678855	-0.857320
H	-1.591943	1.496666	-1.922330
H	-0.496692	2.767137	0.583687
H	0.314729	2.363202	-0.917140
H	-0.150029	-0.298037	2.472171
H	0.559858	1.310331	2.614460
H	-1.128577	1.128419	2.207796
H	0.070635	-0.315210	-1.308250
H	1.888434	0.806940	-0.778570
H	1.648452	-1.284858	2.544696
H	2.830739	-0.021092	2.862258
H	3.356255	-1.646665	2.410841
H	2.511212	-2.630434	0.186976
H	2.231626	-1.544274	-1.167065
H	0.279096	-2.115801	1.135458
H	0.165246	-2.660613	-0.517247
H	2.757022	1.930609	1.900977
H	1.866892	2.915615	0.760579
H	5.706176	2.100525	-1.746536
H	5.093137	0.506162	-2.309869
H	6.391845	0.532291	-1.174234
H	4.689215	-2.312597	0.590865
H	6.060196	-1.280802	0.172565
H	5.167473	-2.601106	-1.645883
H	4.549337	0.109457	1.335512
H	-4.440837	-0.109679	1.657353
H	-6.429555	-1.145127	1.095786

0 imaginary frequencies
E = -1128.791431
G = -1128.204386

TS: 1,2-Hydride Shift from C-18 to C-17 in *β*-C

C	-2.491642	-1.698099	0.726028
C	-1.890749	-0.785811	-0.372690
C	-2.583762	0.610647	-0.222094
C	-4.144335	0.653397	-0.355387
C	-4.685939	-0.359748	0.695152
C	-4.029907	-1.746418	0.644904
C	-1.783312	1.665637	-0.998930
C	-0.425686	1.945635	-0.304155
C	0.206237	0.710371	0.436273
C	-0.318779	-0.581639	-0.270632
C	1.759567	0.759633	0.183947
C	2.593484	-0.503051	0.601962
C	1.945126	-1.754790	-0.073022
C	0.434172	-1.833725	0.203231
C	2.460334	2.040229	0.664609
C	3.787783	2.203104	-0.077589
C	4.612885	0.929671	-0.172530
C	4.023542	-0.341876	0.085069
C	4.895560	-1.578902	-0.135177
O	4.981717	-1.689611	-1.548997
C	2.741293	-0.772503	2.131099
C	5.962904	1.090813	-0.846779

C	-0.110869	0.792471	1.951593
C	-2.101593	-1.459223	-1.755047
C	-4.644905	2.056849	0.067826
C	-4.706252	0.380053	-1.767648
O	-6.101785	-0.458145	0.520191
H	4.409675	2.989704	0.363630
H	3.606933	2.512222	-1.116495
H	-3.092500	-1.891524	-1.876957
H	-1.386520	-2.279401	-1.886779
H	-1.946170	-0.757074	-2.580006
H	-2.103201	-2.720442	0.647929
H	-2.198742	-1.325813	1.716981
H	-4.356853	-2.259602	-0.265388
H	-4.412941	-2.345705	1.481792
H	-4.445785	2.809006	-0.701509
H	-4.174547	2.391417	1.000993
H	-5.725950	2.026708	0.225663
H	-4.647157	-0.665460	-2.069092
H	-4.175888	0.979790	-2.515550
H	-5.761700	0.659616	-1.802531
H	-2.450112	0.868145	0.834594
H	-2.330127	2.608431	-1.075776
H	-1.615915	1.331949	-2.029579
H	-0.556863	2.760191	0.418030
H	0.270725	2.323073	-1.063624
H	0.042929	-0.151593	2.477745
H	0.508237	1.550783	2.442522
H	-1.145234	1.084019	2.130125
H	-0.006509	-0.434888	-1.316155
H	1.842626	0.769411	-0.914445
H	1.809416	-1.144811	2.551488
H	3.024631	0.127329	2.684187
H	3.502711	-1.537422	2.322263
H	2.417292	-2.673791	0.288561
H	2.106321	-1.709781	-1.156640
H	0.251877	-2.024609	1.266225
H	0.061924	-2.718895	-0.322311
H	2.632890	2.025708	1.746824
H	1.844898	2.920904	0.470459
H	6.461483	1.989509	-0.477733
H	5.779194	1.219832	-1.918546
H	6.628372	0.236076	-0.736955
H	4.437234	-2.457017	0.321273
H	5.884833	-1.442250	0.321968
H	5.115801	-2.612937	-1.804049
H	4.776331	0.518170	0.980505
H	-4.476012	0.077822	1.686935
H	-6.478009	-0.981107	1.239794

1 imaginary frequency
E = -1128.780533
G = -1128.194071

***β*-D**

C	-2.516575	-1.629061	0.850527
C	-1.917098	-0.803105	-0.315841
C	-2.570733	0.618333	-0.241575
C	-4.131769	0.694068	-0.344751
C	-4.675356	-0.234160	0.780513
C	-4.057168	-1.639239	0.805031
C	-1.758374	1.596774	-1.100077
C	-0.388720	1.888127	-0.438535
C	0.243434	0.678754	0.345685

C	-0.335816	-0.638769	-0.262572
C	1.792949	0.696729	0.048559
C	2.603666	-0.589187	0.476194
C	1.888243	-1.861472	-0.047902
C	0.388092	-1.878775	0.282367
C	2.514136	1.979951	0.489457
C	3.927228	2.047003	-0.085880
C	4.782797	0.804589	0.277612
C	4.038182	-0.472200	0.079817
C	4.760520	-1.635485	-0.529097
O	4.520500	-1.139971	-1.864541
C	2.839619	-0.774637	2.040660
C	6.207012	0.891314	-0.295967
C	-0.022337	0.842861	1.863720
C	-2.178012	-1.555846	-1.647537
C	-4.585630	2.135209	-0.003049
C	-4.733262	0.346410	-1.723992
O	-6.096575	-0.304821	0.641689
H	4.454386	2.933784	0.277957
H	3.893012	2.124163	-1.179663
H	-3.182111	-1.968115	-1.719769
H	-1.487677	-2.402061	-1.743598
H	-2.025667	-0.909972	-2.517642
H	-2.158856	-2.665123	0.827063
H	-2.191485	-1.206785	1.810907
H	-4.418275	-2.198293	-0.064327
H	-4.436821	-2.174190	1.685671
H	-4.382990	2.831380	-0.822603
H	-4.087317	2.515738	0.897419
H	-5.663515	2.145267	0.176904
H	-4.711042	-0.717842	-1.957395
H	-4.204028	0.880702	-2.520654
H	-5.780967	0.653975	-1.754412
H	-2.407206	0.942241	0.792790
H	-2.281992	2.546356	-1.233460
H	-1.612923	1.191590	-2.108503
H	-0.501427	2.731700	0.252534
H	0.300138	2.228804	-1.221126
H	0.268318	-0.029811	2.451678
H	0.516777	1.707048	2.263485
H	-1.077175	1.020891	2.068496
H	-0.044186	-0.573272	-1.322591
H	1.856606	0.675088	-1.050717
H	1.969004	-1.284847	2.451973
H	2.976966	0.179888	2.546950
H	3.705822	-1.410941	2.261115
H	2.356186	-2.764546	0.359828
H	1.980627	-1.907989	-1.142250
H	0.235588	-1.980859	1.362227
H	-0.029567	-2.789080	-0.158641
H	2.554215	2.061247	1.581572
H	1.953540	2.852874	0.145673
H	6.616249	1.875249	-0.054897
H	6.206169	0.785606	-1.383574
H	6.887637	0.151083	0.132622
H	4.322228	-2.615103	-0.351550
H	5.833499	-1.659171	-0.356259
H	3.745060	-1.579594	-2.247750
H	4.874897	0.812370	1.383537
H	-4.431400	0.260027	1.737318
H	-6.471872	-0.760265	1.406270

0 imaginary frequencies
E = -1128.786585
G = -1128.197213

TS: D Ring Cyclization Leading from B to β-C (boat-like TS)

C	-4.553213	-0.031648	-0.379608
C	-2.493413	-0.475054	-0.704517
C	-1.784130	0.814866	-0.334013
C	-2.518181	2.052157	-0.875357
C	-3.765099	2.288358	0.024557
C	-4.373612	1.006437	0.528244
C	-2.083506	-1.616054	0.181252
C	-0.538420	-1.791385	-0.008678
C	0.248577	-0.506130	0.299213
C	-0.209625	0.714160	-0.572597
C	1.813740	-0.744730	0.454695
C	2.552891	0.596958	0.127870
C	1.789831	1.773357	0.756045
C	0.431360	2.007396	0.040743
C	4.113903	0.607026	0.268998
C	4.628868	-0.557864	-0.626401
C	3.928750	-1.902969	-0.389693
C	2.394257	-1.818670	-0.498835
C	1.986168	-1.227835	1.919751
C	4.660164	1.922113	-0.341661
C	4.660129	0.517050	1.710203
O	6.038976	-0.673608	-0.424629
C	0.125226	0.623213	-2.081326
C	-2.675352	-0.862315	-2.159200
C	-4.646339	0.923432	1.988363
C	-5.343967	-1.301651	-0.140231
O	-5.018055	-1.888565	1.112490
H	-4.535079	2.840523	-0.534149
H	-3.492499	2.915689	0.878633
H	2.963861	-1.663458	2.115328
H	1.246917	-2.002888	2.151405
H	1.841066	-0.414198	2.636925
H	1.970905	-2.807069	-0.284049
H	2.122995	-1.579745	-1.536170
H	4.229777	-2.291029	0.588796
H	4.299955	-2.626540	-1.127592
H	4.482516	2.781175	0.312479
H	4.205850	2.137381	-1.317022
H	5.740364	1.836982	-0.484048
H	4.583386	-0.477696	2.149095
H	4.134951	1.220113	2.365819
H	5.719745	0.782004	1.716878
H	2.435388	0.716454	-0.954683
H	2.367471	2.698865	0.701710
H	1.621768	1.588328	1.823223
H	0.564513	2.744012	-0.759856
H	-0.258337	2.462702	0.762038
H	0.011343	-0.382337	-2.489840
H	-0.511727	1.297225	-2.664351
H	1.152131	0.927317	-2.280285
H	-0.067753	-0.213117	1.311399
H	-1.842279	0.880275	0.760112
H	-1.763462	-1.358754	-2.503065
H	-2.862641	-0.017517	-2.823194
H	-3.475766	-1.596759	-2.277328
H	-2.599495	-2.544150	-0.075056
H	-2.287187	-1.398901	1.232813
H	-0.329063	-2.155442	-1.019545
H	-0.235541	-2.594024	0.670301
H	-2.806334	1.922985	-1.922633

H	-1.891974	2.945351	-0.837470
H	-5.184766	1.829066	2.298072
H	-3.691760	0.957872	2.536767
H	-5.187355	0.028799	2.284819
H	-5.143699	-2.001543	-0.963884
H	-6.414114	-1.047692	-0.193972
H	-5.591360	-2.650892	1.269004
H	-4.626693	0.301618	-1.412141
H	4.437581	-0.256432	-1.671302
H	6.404987	-1.298463	-1.063780

1 imaginary frequency
E = -1128.785366
G = -1128.198115

TS: 1,2-Methyl Shift from C-13 to C-18 in β-D

C	2.470619	-1.695351	-0.748187
C	1.945764	-0.787250	0.393123
C	2.635232	0.606354	0.210796
C	4.201375	0.640253	0.238037
C	4.666665	-0.365471	-0.854995
C	4.010457	-1.749993	-0.768333
C	1.895967	1.654473	1.053032
C	0.499698	1.952680	0.452168
C	-0.201693	0.728884	-0.241859
C	0.368299	-0.579849	0.396174
C	-1.737976	0.807958	0.122439
C	-2.575589	-0.455639	-0.178862
C	-1.892159	-1.740211	0.292809
C	-0.405953	-1.819732	-0.075975
C	-2.449805	2.097162	-0.314859
C	-3.882464	2.131056	0.212256
C	-4.746193	0.961268	-0.295800
C	-3.997096	-0.378095	-0.246208
C	-3.015008	-0.589360	-1.895680
C	-4.847888	-1.641622	-0.060211
O	-5.099572	-1.748924	1.332684
C	-6.098256	0.939102	0.453123
C	-0.007933	0.801836	-1.776880
C	2.240701	-1.475506	1.753148
C	4.677100	2.045896	-0.206281
C	4.857030	0.351671	1.606136
O	6.089885	-0.469617	-0.776613
H	-4.372874	3.067715	-0.070088
H	-3.870172	2.107351	1.309937
H	3.237639	-1.907106	1.810329
H	1.535598	-2.298754	1.918223
H	2.134762	-0.782959	2.593813
H	2.085811	-2.717487	-0.650074
H	2.115261	-1.318370	-1.716784
H	4.394282	-2.269408	0.115778
H	4.334414	-2.346759	-1.631437
H	4.534692	2.791989	0.581217
H	4.145626	2.390606	-1.102150
H	5.744592	2.013554	-0.438190
H	4.831624	-0.699614	1.892062
H	4.370937	0.931941	2.397939
H	5.909117	0.643485	1.575856
H	2.431891	0.879536	-0.831317
H	2.452127	2.593325	1.105955
H	1.794571	1.308708	2.088414
H	0.591573	2.764667	-0.278602
H	-0.138678	2.340191	1.255207
H	-0.368477	-0.088966	-2.298275
H	-0.521444	1.671703	-2.198201
H	1.041780	0.911933	-2.045096
H	0.119504	-0.468222	1.462803
H	-1.757752	0.808934	1.224969
H	-2.351801	-1.414619	-2.148423
H	-2.731934	0.359470	-2.336300
H	-4.007734	-0.865170	-2.262691
H	-2.412293	-2.633026	-0.062693
H	-1.984096	-1.734040	1.387911
H	-0.290278	-1.970482	-1.156006
H	-0.001819	-2.722146	0.392025
H	-2.450603	2.208368	-1.405701
H	-1.900210	2.959496	0.069006
H	-6.531149	1.940885	0.387034

```
H   -5.971344    0.689580    1.508356
H   -6.822748    0.245985    0.021313
H   -4.357240   -2.529351   -0.463758
H   -5.805174   -1.530647   -0.574797
H   -4.574623   -2.461969    1.719357
H   -4.978358    1.138904   -1.356974
H    4.390310    0.079679   -1.827043
H    6.416818   -0.983671   -1.526131
1 imaginary frequency
E = -1128.782783
G = -1128.193669
```

Carbocation product of the 1,2-Methyl Shift from C-13 to C-18 in β-D

```
C    2.398331   -1.725822   -0.675032
C    1.995921   -0.772840    0.478475
C    2.620105    0.625208    0.148697
C    4.178188    0.687995   -0.013206
C    4.535029   -0.362528   -1.105711
C    3.925535   -1.752993   -0.878190
C    1.963310    1.698560    1.026542
C    0.499320    1.947650    0.581175
C   -0.230498    0.669073    0.052862
C    0.419029   -0.597956    0.674576
C   -1.781786    0.740936    0.613795
C   -2.497589   -0.491810    0.272778
C   -1.868475   -1.747883    0.749031
C   -0.369478   -1.862535    0.331006
C   -2.496056    2.077728    0.330274
C   -4.008294    1.921533    0.184803
C   -4.335050    0.871522   -0.886590
C   -3.794574   -0.545822   -0.486291
C   -3.583996   -1.404191   -1.780243
C   -4.790647   -1.344590    0.417107
O   -4.808908   -0.739553    1.705793
C   -5.827107    0.897192   -1.252559
C   -0.286995    0.647960   -1.489684
C    2.470601   -1.384446    1.822576
C    4.562431    2.078309   -0.577302
C    4.998406    0.481626    1.279381
O    5.958763   -0.437955   -1.188929
H   -4.442381    2.883794   -0.106269
H   -4.467053    1.636327    1.137568
H    3.483652   -1.776716    1.775286
H    1.823274   -2.221997    2.106549
H    2.438823   -0.655428    2.637751
H    2.051194   -2.748029   -0.482831
H    1.921669   -1.402553   -1.610553
H    4.424552   -2.222967   -0.024741
H    4.156915   -2.384146   -1.746157
H    4.498395    2.860156    0.185481
H    3.920451    2.369294   -1.418257
H    5.594997    2.053513   -0.934070
H    5.029863   -0.552527    1.622760
H    4.599925    1.098390    2.092278
H    6.033122    0.786091    1.107172
H    2.287718    0.845793   -0.873808
H    2.495951    2.650521    0.968288
H    1.990784    1.402830    2.081268
H    0.486389    2.705568   -0.209469
H   -0.046959    2.381760    1.423722
H   -0.737844   -0.269602   -1.880953
H   -0.860179    1.492977   -1.880335
H    0.711193    0.726532   -1.920603
```

```
H    0.302775   -0.451369    1.757354
H   -1.617004    0.645425    1.698258
H   -2.416450   -2.636516    0.438715
H   -1.906941   -1.708427    1.847708
H   -0.318015   -2.092890   -0.736727
H    0.032655   -2.733530    0.854904
H   -2.099327    2.530960   -0.583222
H   -2.261848    2.773814    1.140284
H   -6.068287    1.864460   -1.702753
H   -6.464716    0.785177   -0.369821
H   -6.104732    0.128860   -1.981148
H   -4.482140   -2.394168    0.482689
H   -5.774006   -1.313202   -0.061639
H   -5.496504   -1.143154    2.252956
H   -3.779906    1.153620   -1.792949
H    4.137644    0.029653   -2.058504
H    6.210351   -0.970109   -1.954773
H   -4.548247   -1.495714   -2.285995
H   -2.894113   -0.912259   -2.468531
H   -3.218427   -2.411397   -1.571396
0 imaginary frequencies
E = -1128.808985
G = -1128.219316
```

References

[1] V. L. Challinor, S. Chap, R. P. Lehmann, P. V. Bernhardt and J. J. De Voss, *J. Nat. Prod.* 2013, **76**, 485–488.

[2] H. Takikawa and M. Sugiyama, Jpn. Kokai Tokkyo Koho JP 2012206962, 2012.

[3] G. Brownlie and F. S. Spring, R. Stevenson, W. S. Strachan, *J. Chem. Soc. (Resumed)* 1956, 2419-2427.

[4] G. M. Sheldrick, *Acta Crystallogr., Sect. A: Found. Crystallogr.* 2008, **A64**, 112-122.

[5] L. J. Farrugia, *J. Appl. Crystallogr.* 1999, **32**, 837-838.

[6] L. J. Farrugia, *J. Appl. Crystallogr.* 1997, **30**, 565.

[7] R. W. W. Hooft, L. H. Straver and A. L. Spek, *J. Appl. Crystallogr.* 2008, **41**, 96-103.

[8] M. J. Frisch, G. W. Trucks, H. B. Schlegel, G. E. Scuseria, M. A. Robb, J. R. Cheeseman, G. Scalmani, V. Barone, B. Mennucci, G. A. Petersson, H. Nakatsuji, M. Caricato, X. Li, H. P. Hratchian, A. F. Izmaylov, J. Bloino, G. Zheng, J. L. Sonnenberg, M. Hada, M. Ehara, K. Toyota, R. Fukuda, J. Hasegawa, M. Ishida, T. Nakajima, Y. Honda, O. Kitao, H. Nakai, T. Vreven, J. A. Montgomery, Jr., J. E. Peralta, F. Ogliaro, M. Bearpark, J. J. Heyd, E. Brothers, K. N. Kudin, V. N. Staroverov, R. Kobayashi, J. Normand, K. Raghavachari, A. Rendell, J. C. Burant, S. S. Iyengar, J. Tomasi, M. Cossi, N. Rega, J. M. Millam, M. Klene, J. E. Knox, J. B. Cross, V. Bakken, C. Adamo, J. Jaramillo, R. Gomperts, R. E. Stratmann, O. Yazyev, A. J. Austin, R. Cammi, C. Pomelli, J. W. Ochterski, R. L. Martin, K. Morokuma, V. G. Zakrzewski, G. A. Voth, P. Salvador, J. J. Dannenberg, S. Dapprich, A. D. Daniels, Ö. Farkas, J. B. Foresman, J. V. Ortiz, J. Cioslowski, D. J. Fox, Gaussian 09, Revision D.01, Gaussian, Inc., Wallingford CT, 2013.

[9] A. D. Becke, *J. Chem. Phys.* 1993, **98**, 5648-5652.

[10] S. Miertuš, E. Scrocco and J. Tomasi, *Chem. Phys.* 1981, **55**, 117-129.

[11] C. Adamo and V. Barone, *J. Chem. Phys.*, 1998, **108**, 664-675.

[12] a) W. J. Hehre, R. Ditchfield and J. A. Pople, *J. Chem. Phys.* 1972, **56**, 2257-2261; b) P. C. Hariharan and J. A. Pople, *Theor. Chim. Acta* 1973, **28**, 213-222.

[13] a) M. W. Lodewyk, C. Soldi, P. B. Jones, M. M. Olmstead, J. R. Larrucea, J. T. Shaw and D. J. Tantillo, *J. Am. Chem. Soc.* 2012, **134**, 18550-18553; b) Scripts, test cases and other miscellany can be found at the Chemical Shift Repository http://cheshirenmr.info/index.htm.

[14] a) S. G. Smith and J. M. Goodman, *J. Am. Chem. Soc.* 2010, **132**, 12946-12959; b) A Java applet for calculation of the DP4 probability can be found at: Assignment of stereochemistry and structure using NMR and DP4, http://www-jmg.ch.cam.ac.uk/tools/nmr/DP4/

[15] P. J. Stephens and D. M. McCann, J. R. Cheeseman, M. J. Frisch, *Chirality* 2005, **17**, S52-S64.

[16] a) C. Gonzalez and H. B. Schlegel, *J. Chem. Phys.* 1989, **90**, 2154-2161; b) C. Gonzalez and H. B. Schlegel, *J. Phys. Chem.* 1990, **94**, 5523-5527.
